# Systematic review and meta‐analysis identify significant relationships between clinical anxiety and lower urinary tract symptoms

**DOI:** 10.1002/brb3.2268

**Published:** 2021-08-17

**Authors:** Behrang Mahjani, Lotta Renström Koskela, Anita Batuure, Christina Gustavsson Mahjani, Magdalena Janecka, Christina M. Hultman, Abraham Reichenberg, Joseph D. Buxbaum, Olof Akre, Dorothy E. Grice

**Affiliations:** ^1^ Department of Psychiatry Icahn School of Medicine at Mount Sinai New York USA; ^2^ Seaver Autism Center for Research and Treatment Icahn School of Medicine at Mount Sinai New York USA; ^3^ Department of Medical Epidemiology and Biostatistics Karolinska Institutet Stockholm Sweden; ^4^ Department of Genetics and Genomic Sciences Icahn School of Medicine at Mount Sinai New York USA; ^5^ Department of Neuroscience Icahn School of Medicine at Mount Sinai New York USA; ^6^ The Mindich Child Health and Development Institute Icahn School of Medicine at Mount Sinai New York USA; ^7^ Division of Tics, OCD, and Related Disorders Icahn School of Medicine at Mount Sinai New York USA; ^8^ Friedman Brain Institute Icahn School of Medicine at Mount Sinai New York USA; ^9^ Department of Molecular Medicine and Surgery Karolinska Institutet Stockholm Sweden; ^10^ Department of Pelvic Cancer Karolinska University Hospital Stockholm Sweden

**Keywords:** anxiety disorders, interstitial cystitis, lower urinary tract symptoms, obsessive‐compulsive disorder, overactive bladder

## Abstract

**Background:**

Lower urinary tract symptoms (LUTS), such as voiding symptoms, overactive bladder, and interstitial cystitis, and anxiety disorders are often comorbid conditions in patients. However, the existing evidence regarding the rates and nature of the co‐occurrence of these conditions has not been systematically evaluated. The aim of this study was to examine these relationships.

**Methods:**

We conducted a systematic review and meta‐analysis to examine the relationship between LUTS and anxiety. We searched for articles published from January 1990 to July 2019 in PubMed, CENTRAL, PsycINFO, and Google Scholar. Outcomes were anxiety‐related disorders and symptoms (clinically significant anxiety) and LUTS. We performed random‐effects meta‐analyses, inspected funnel plots, and applied the Egger's test to evaluate publication bias. We followed PRISMA guidelines and recorded our protocol on PROSPERO (ID = CRD42019118607).

**Results:**

We identified 814 articles, of which 94 fulfilled inclusion criteria, and 23 had sufficient data for meta‐analysis. The odds ratio (OR) for clinically significant anxiety among individuals with LUTS was 2.87 (95% CI: 2.38,3.46, *p* < .001). The OR for LUTS among individuals with clinically significant anxiety was 2.87 (95% CI: 1.07,7.74, *p* < .001), although very few studies examined this relationship. A large value of I^2^ index suggests high heterogeneity between studies.

**Conclusion:**

The results demonstrate a significant association between clinically significant anxiety and LUTS in both females and males. There were limited studies on younger individuals and on individuals ascertained for clinically significant anxiety, which should motivate further study in these areas. Understanding the co‐occurrence of these conditions will lead to better prevention and interventions to ameliorate the progression of the symptoms and improve the quality of life. A thorough assessment of anxiety may provide more optimal care for LUTS patients.

## Graphical abstract

The results demonstrate a significant association between clinically significant anxiety and LUTS in both females and males. LUTS is a common disorder in children and adults and clinicians should be aware of these associations because they can inform optimal assessment and treatment for both mental health and medical conditions.

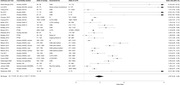

## 1 INTRODUCTION

Anxiety disorders are among the most common psychiatric conditions, with lifetime prevalences as high as 33.7% (Bandelow & Michaelis, 2015). Although it is not uncommon to experience anxiety symptoms over the course of life, when the anxiety persists and impairs daily function, criteria for a clinical diagnosis may be met (Keller et al., 1992).

**FIGURE 1:**
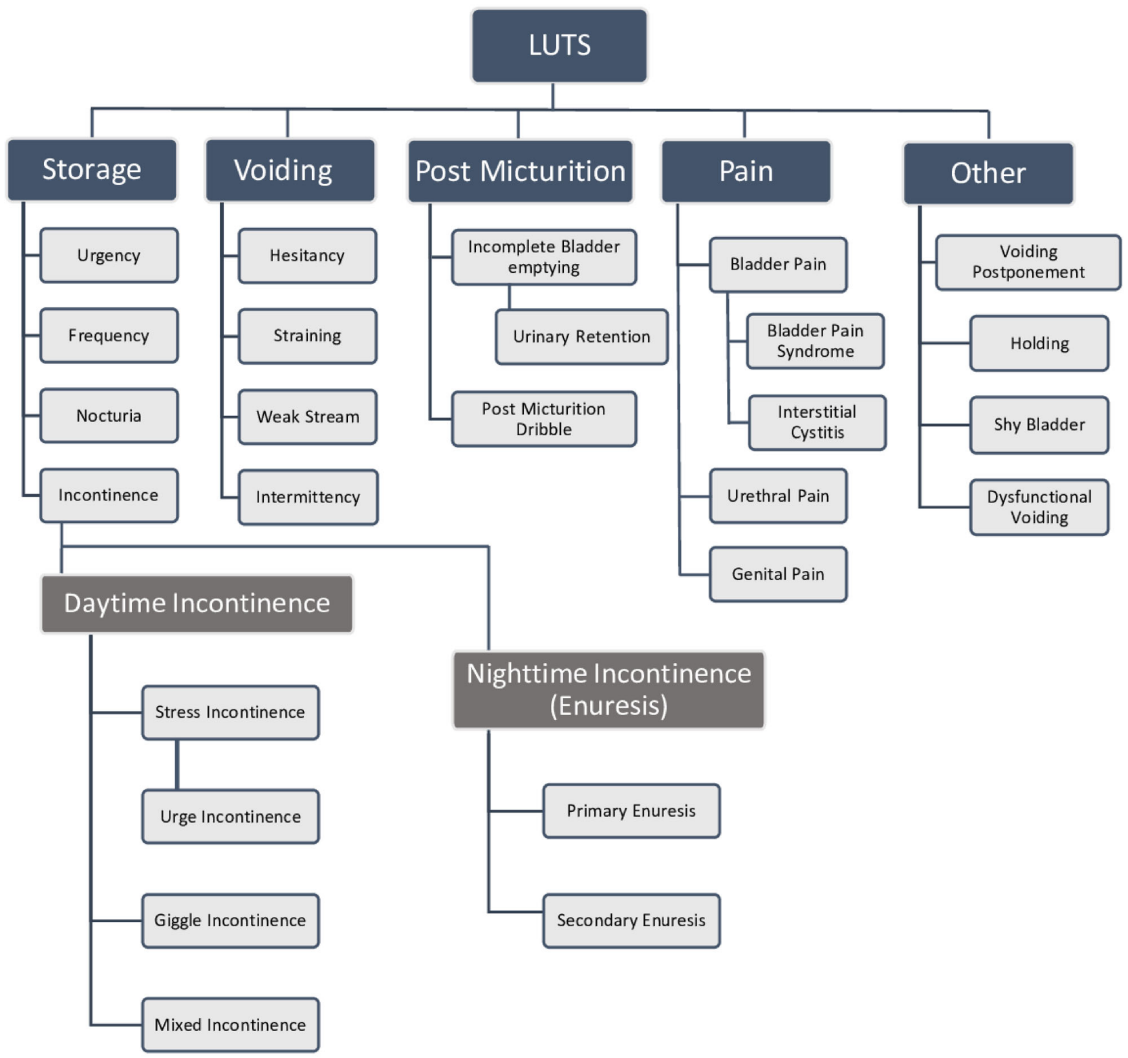
Subcategories of lower urinary tract symptoms (LUTS)

Lower urinary tract symptoms (LUTS) are related to dysfunction of the lower urinary system, including the bladder, prostate, and urethra. LUTS can present across the life cycle in a variety of ways: through issues with storage (urgency, frequency, or incontinence), voiding (hesitancy, straining, intermittency, and weak stream), post micturition (dribble and incomplete emptying; Figure 1), and pain. The potential symptoms arising from the lower urinary tract overlap in adults and children, but the incidence of symptoms, and symptom causes, vary between these groups. The population frequency of LUTS in adults is between 20% and 30% and varies due to definitions of LUTS (Coyne et al., 2009; Soler et al., 2018). LUTS can onset in childhood, and the prevalence increases over the lifespan, with a secondary increase related to consequences of the aging. Here, we focus on LUTS in children, adolescents, and younger adults and avoid LUTS related to aging processes (Boyle et al., 2003).

Specific LUTS symptoms, such as increased urinary frequency, pressure, urgency, and/or pain when the bladder fills, can have significant and direct effects on the overall quality of life (Milsom et al., 2012). The experience of LUTS in youth can put them at risk for social isolation, teasing, and other challenges, and they may experience low self‐esteem or distress (Thibodeau et al., 2013).

Studies in children and adults with LUTS indicate that those with LUTS are more likely to also have anxiety symptoms, anxiety disorders (Huang et al., 2017; for more studies, see Table S1), obsessive‐compulsive disorder (OCD; Ahn et al., 2016; Bernstein et al., 2010; Bogner et al., 2011; Drummond et al., 2012; Frankovich et al., 2015; Hsiao et al., 2014; Jaspers‐Fayer et al., 2017; Macaulay et al., 1991; Murphy & Pichichero, 2002; Özen et al., 2019; Swedo et al., 2015; Talati et al., 2008), attention‐deficit/hyperactivity disorder (ADHD; Mahjani et al., 2021). Of note, a large longitudinal population‐based cohort study argues that there is a bidirectional improvement when treating either LUTS or psychiatric symptoms (Huang et al., 2017). To date, no meta‐analyses of associations between anxiety disorders and LUTS have been published. We hypothesis that there is a strong association between anxiety disorders, anxiety symptoms, and LUTS. In order to shed light on these associations, we conducted a systematic review and meta‐analysis, examining studies in both children and adults.

## 2 MATERIAL AND METHODS

### 2.1 Search strategy and selection criteria

**FIGURE 2:**
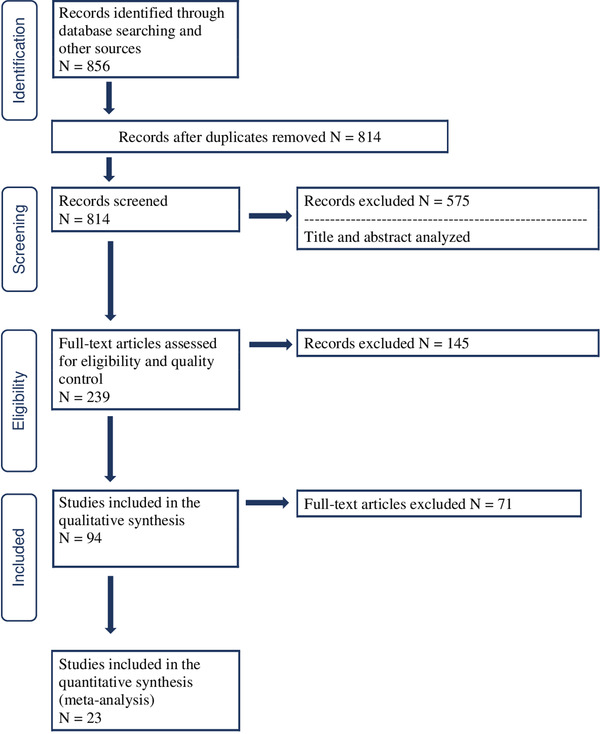
PRISMA details

We recorded our approach using the Preferred Reporting Items for Systematic Reviews and Meta‐Analyses (PRISMA) protocol (Moher et al., 2009; Supplement 1) in International Prospective Register of Systematic Reviews (PROSPERO) (23) (ID = CRD42019118607). Figure 2 outlines the steps used to search the literature for qualifying articles. We summarize the overall approach here and provide greater detail further in the Methods section. (1) *Identification*: We identified all articles with results in humans that included our search terms referencing anxiety disorders or LUTS in the title OR abstract, and anxiety disorders AND LUTS in the main text (Supplement 2); (2) *Screening*: At least two authors (BM, MJ, AB) independently screened all identified articles for inclusion/exclusion criteria, based on titles and abstracts, and 20 articles were randomly chosen and re‐screened by a third author (DEG); (3) *Eligibility*: At least two authors (CGM, AB, BM) independently reviewed the full text of each selected article and applied the exclusion criteria. Excluded articles and conflicts were reviewed by a different author and, again, 20 articles were randomly chosen and re‐screened by DEG. We excluded studies with sample sizes of less than 20 individuals, samples where all individuals were older than 50 years (due to the impact of aging on urinary symptoms), articles reporting only nocturnal enuresis, or when it was not possible to separate the study results for daytime versus nighttime wetting. We also excluded treatment trials because it was not possible to discern if clinical symptoms were secondary to the intervention or independent of it.

We searched systematically for articles in PubMed, CENTRAL, PsycINFO, and Google Scholar (specific search terms in Supplement 2). We used Google Scholar to find articles that might have been missed by the searches via PubMed, CENTRAL, and PsycINFO. We performed 12 searches in Google Scholar using different keywords (details in Supplement 2). For each search, we screened the first 100 articles and identified only a single article that was not found previously (details in Supplement 2). We also examined all references from relevant review articles to find any eligible articles to complement the database searches.

Observational studies were included in the review (cohort, case‐control, and cross‐sectional studies). We did not include ecological, treatment, or case series studies. We considered peer‐reviewed, published studies (including studies published online ahead of print or online only) in English (or translated into English) from January 1, 1990, to July 15, 2019. We excluded all studies published as editorials or commentaries. We did not seek data from any unpublished studies.

For each manuscript, we checked the quality of study design, the power of the study, and the use of appropriate statistical techniques. Each article review was carried out by at least two individuals (MJ, BM, AB, CGM) who independently utilized a checklist of clinical parameters, methodology, and results to determine eligibility for inclusion. Any conflicts were discussed until a consensus was reached. A second‐tier review was performed by experts in child psychiatry, urology, and statistics (DEG, LRK, OA, JDB) who assessed study summaries and checklists, and final inclusion was determined by expert consensus.

### 2.2 Psychiatric outcomes

Anxiety disorders cover a broad spectrum of conditions. Generalized anxiety disorder (GAD), social anxiety disorder, separation anxiety disorder, panic disorder (PD), and specific phobias occur across the lifespan, with significant rates in children, adolescents, and adults 1994, 2013 (Coyne et al., 2009; Soler et al., 2018). Selective mutism is largely described in younger school‐age children, but it can continue through childhood, particularly in the absence of treatment. Although OCD and posttraumatic stress disorder (PTSD) were moved from the anxiety disorder section of Diagnostic and Statistical Manual of Mental Disorders, fourth edition (DSM IV), to separate sections in DSM fifth edition (DSM 5), albeit with minimal changes in diagnostic criteria, anxiety symptoms are also at the core of these conditions (American Psychiatric Association, 1994; American Psychiatric Association, 2013). Pediatric acute‐onset neuropsychiatric syndrome (PANS) is characterized by an abrupt onset of obsessive‐compulsive symptoms or eating restrictions. When associated with streptococcal infection, the condition is subtyped as a pediatric autoimmune neuropsychiatric disorder associated with streptococcal infections (PANDAS; Hsiao et al., 2014; Macaulay et al., 1991; Özen et al., 2019; Swedo et al., 2015). In the trauma‐ and stressor‐related section of DSM 5, the diagnosis of PTSD requires exposure to a life‐threatening trauma, which can occur at any time across the life cycle. The psychiatric sequelae of PTSD are manifest by severe anxiety symptoms, albeit brought on an external exposure, unlike the anxiety disorders described above.

In this study, we used the following psychiatric outcomes: GAD, social anxiety disorder, separation anxiety, adjustment disorder, selective mutism, PD, agoraphobia, phobias, OCD, and PTSD. We also included all possible cases of anxiety disorders based on elevated anxiety symptoms, even where the specific reference to International Classification of Diseases (ICD)/DSM was missing, provided the study participants obtained a clinically significant result on standardized questionnaires/measures. Standardized questionnaires, such as Hospital Anxiety and Depression Scale (HADS), Zung Self‐Rating Anxiety Scale (SAS), and GAD 7‐item do not include all of the diagnostic criteria of anxiety disorders and are primarily designed to measure anxiety symptoms and detect possible causes. In this work, we defined *clinically significant anxiety* as having an anxiety‐related disorder or elevated anxiety symptoms based on standardized questionnaires.

### 2.3 Urological outcomes

Urinary incontinence (UI) can be divided into subgroups, and in many publications, authors make a distinction between enuresis/nocturnal enuresis and daytime UI (Figure 1). However, in both ICD‐10 and DSM 5, enuresis and daytime incontinence are coded as one disorder, without differentiating between subtypes of incontinence (von Gontard & Equit, 2014). Enuresis is defined as any intermittent incontinence while sleeping (at night or during daytime naps). Daytime incontinence includes: urge incontinence, stress incontinence, and mixed incontinence (Figure 1; Austin et al., 2016).

Overactive bladder (OAB) is a complex of storage symptoms with frequency, urgency, nocturia, and in some patients, incontinence. OAB can occur at any age, although the prevalence of the different symptoms varies across age groups (McLennan, 2014). Interstitial cystitis (IC) and bladder pain syndrome are characterized by lower abdominal pain and pressure upon filling of the bladder that has lasted for more than 6 months and is accompanied by at least one additional symptom, such as frequency or urgency. IC is a subcategory of bladder pain syndrome characterized by an active inflammation of the bladder. IC and OAB have similar symptoms, but pelvic pain, which is a pathognomonic symptom of IC, is generally not present in patients with OAB (Macdiarmid & Sand, 2007) and has been rarely reported among children (Close et al., 1996).

In this study, we used the following urological outcomes: OAB, bladder pain syndrome, IC, urinary storage symptoms, urinary voiding symptoms, and daytime UI. We excluded LUTS associated with physical trauma, cancer, sexually transmitted infection, congenital malformation of the urinary tract, post‐operative disorders, those related primarily to pregnancy, dysfunction secondary to aging, and medications that promote urination/water retention. We excluded outcomes associated with kidney malfunction unless they arose as a consequence of a lower urinary tract infection. We included LUTS due to infection, as regulation of the immune system could be a potential common factor linking urinary and psychiatric symptoms.

### 2.4 Statistical analysis

For the meta‐analysis, we extracted the number of exposed/unexposed individuals with LUTS or clinically significant anxiety from the published articles to calculate the odds ratio (OR). We excluded articles that did not provide the data required for meta‐analysis. If multiple articles analyzed the same cohort, then we chose only one article. The screening was completed using DistillerSR (Evidence Partners).

Utilizing data from eligible studies, we performed meta‐analyses, employing random effects models using reciprocal of the estimated variance, allowing for combining effect estimates without access to raw data. We reported two measures of heterogeneity, the Cochran's Q test, and the I^2^ index. To evaluate publication bias, we visually inspected funnel plots and applied Egger's test (Egger et al., 1997; Schwarzer et al., 2015). We used the *metafor* package in R for the meta‐analysis (Viechtbauer, 2010).

We did two sensitivity analyses to shed light on the heterogeneity of the results. (1) We dissected the outcomes for clinically significant anxiety into anxiety disorders based on ICD codes, anxiety symptoms based on HADS, and anxiety symptoms based on other scales; (2) We dissected the outcomes for LUTS into IC, OAB, UI, and other LUTS.

## 3 RESULTS

We identified 814 articles published from January 1, 1990, to July 15, 2019, that met the inclusion criteria (Figure 2, identification). Following the review of titles and abstracts, and then full texts, 94 articles were included in the qualitative and quantitative syntheses. The characteristics of these articles are summarized in Table S1. Twenty‐three articles were included in the meta‐analysis and are summarized in Table S2.

There were two striking initial findings. First, there was only one study in pediatric clinically significant anxiety with sufficient data for inclusion in the meta‐analysis (Joinson et al., 2006). Second, the overwhelming majority of studies suitable for inclusion in the meta‐analysis involved individuals first ascertained with a LUTS diagnosis, who were then evaluated for anxiety phenotypes (*n* = 21). Only two studies suitable for meta‐analysis examined individuals ascertained for clinically significant anxiety who were then assessed for LUTS (Huang et al., 2017; Talati et al., 2008). Hence, beyond a perfunctory understanding of these relationships, there are clear gaps in the literature.

### 3.1 Clinically significant anxiety among individuals diagnosed with LUTS

The largest group of qualifying studies were those examining anxiety disorders, or their proxy, in individuals ascertained with LUTS, 21 of which had sufficient data for meta‐analysis. Two studies included several anxiety diagnoses, and we selected the larger clinical category: Using data from the Avon Longitudinal Study of Parents and Children, Joinson et al. (2006) included separation anxiety, social fears, and general anxiety, and we chose general anxiety because it had the largest number of subjects; Talati et al. (2008) included both PD and social anxiety disorder, and we chose the PD category, which was larger.

**FIGURE 3:**
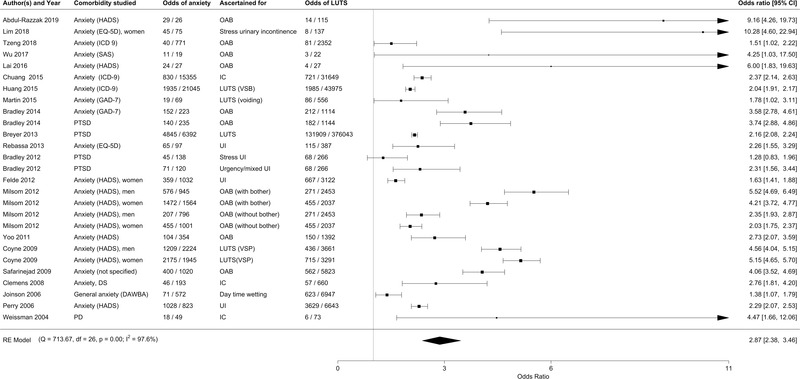
Forest plot for clinically significant anxiety among individuals with LUTS. PTSD: posttraumatic stress disorder, PD: panic disorder, OAB: overactive bladder, IC: interstitial cystitis, UI: urinary incontinence, LUTS: lower urinary tract symptoms, VSP: voiding, storage, postmicturition, VSB: voiding, storage, benign prostatic hyperplasia, EQ‐5D: EuroQol: standardized instrument for measuring generic health status, HADS: Hospital Anxiety and Depression Scale, SAS: The Zung Self‐Rating Anxiety Scale, GAD‐7: General Anxiety Disorder 7‐item, DAWBA: Development And Well‐Being Assessment, ICD 9: International Classification of Diseases code version 9, DS: dissociative and somatoform disorders. *p* is the *p*‐value for I^2^ index

Using a random‐effects model, the OR for clinically significant anxiety among cases with LUTS was 2.87 (95% confidence interval (CI): 2.38,3.46, *p* < .001; Figure 3). When we excluded PTSD from the core anxiety group, the OR for the association between clinically significant anxiety and LUTS was 3.01 (95% CI: 2.44,3.71, *p* < .001; Figure S1).

A few studies used data from the Longitudinal Health Insurance Database 2000 in Taiwan (LHID2000) (Chuang et al., 2015; Huang et al., 2015, 2017; Tzeng et al., 2019). LHID2000 includes the claims data of one million individuals (approximately 5% of the population) randomly sampled from the registry of the National Health Insurance Research Database in 2000. In these studies, the OR for clinically significant anxiety among cases with LUTS was between 1.51 and 2.37 (Figure 3).

**FIGURE 4:**
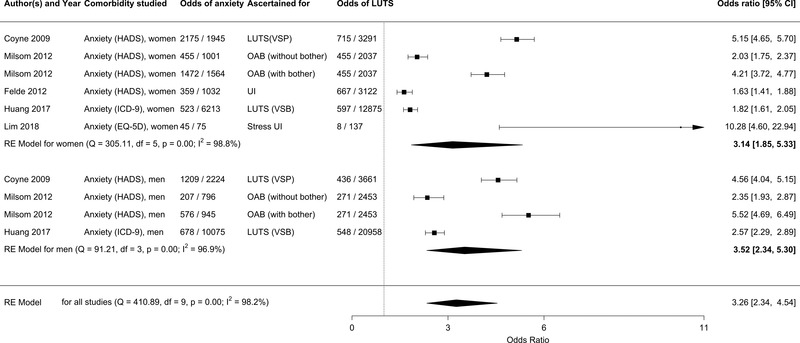
Forest plot for clinically significant anxiety among individuals with LUTS, comparing men and women. OAB: overactive bladder, UI: urinary incontinence, LUTS: lower urinary tract symptoms, VSP: voiding, storage, postmicturition, VSB: voiding, storage, benign prostatic hyperplasia, EQ‐5D: EuroQol: standardized instrument for measuring generic health status, HADS: Hospital Anxiety and Depression Scale, ICD 9: International Classification of Diseases code version 9. *p* is the *p*‐value for I^2^ index

Because the rates of LUTS differ between men and women (Maserejian et al., 2013), we also examined the qualifying articles for differential risk as a function of sex. Few studies qualified for this sub‐analysis. Among these studies, the OR for clinically significant anxiety among women with LUTS was 3.14 (95% CI: 1.85,5.3, *p* < .001), and for men it was 3.52 (95% CI: 2.34,5.30, *p* < .001; Figure 4).

Of the 94 articles that investigated an association between clinically significant anxiety and LUTS, 16 articles focused on children (Bernstein et al., 2010; Filce & Lavergne, 2013; Frankovich et al., 2015; Jaspers‐Fayer et al., 2017; Joinson et al., 2006; Kuizenga‐Wessel et al., 2018; Murphy & Pichichero, 2002; Niemczyk et al., 2018; Oliver et al., 2013; Özen et al., 2019; Schast Stephen et al., 2008; Swedo et al., 2015; von Gontard et al., 1998; Von Gontard et al., 2015; Wolfe‐Christensen et al., 2012; Zink et al., 2008), and only one article had the required information for meta‐analysis (Joinson et al., 2006). Hence, we could not perform a child‐specific meta‐analysis. However, our qualitative review of these studies shows that between 1% and 20% of children with LUTS had clinically significant anxiety (Joinson et al., 2006; Kuizenga‐Wessel et al., 2018; Niemczyk et al., 2018; Oliver et al., 2013; Özen et al., 2019; Schast Stephen et al., 2008; Wolfe‐Christensen et al., 2012; Zink et al., 2008), and 19%–46% had at least one psychiatric comorbidity, with ADHD having the highest rate (Niemczyk et al., 2018; Oliver et al., 2013; Schast Stephen et al., 2008; von Gontard et al., 1998; Wolfe‐Christensen et al., 2012; Zink et al., 2008).

### 3.2 LUTS in individuals diagnosed with clinically significant anxiety

Only two articles (Huang et al., 2017; Talati et al., 2008) had sufficient data for meta‐analysis of LUTS in individuals ascertained with clinically significant anxiety. The OR was 2.87 (95% CI: 1.07,7.74, *p* = .037; Figure S2), similar to the studies in which individuals were ascertained for LUTS.

### 3.3 Bidirectional studies

In a study of the association between anxiety disorders (anxiety states, phobic disorders, obsessive‐compulsive disorders, and adjustment disorder with anxiety) and LUTS, after controlling for age, sex, and medical comorbidities, individuals with LUTS were 2.12 (95% CI: 1.95–2.30) times more likely to develop anxiety disorders, and individuals with anxiety disorders were 2.01 (95%CI: 1.88–2.14) times more likely to develop LUTS (Huang et al., 2017). In another study, UI and frequency were predictors of incident cases of anxiety (measured using HADS), and anxiety was a predictor of incident cases of UI (Perry et al., 2006).

### 3.4 Association of LUTS and OCD

We were also interested in the specific relationship between OCD and LUTS. We found 12 articles discussing this association (Ahn et al., 2016; Bernstein et al., 2010; Bogner et al., 2011; Drummond et al., 2012; Frankovich et al., 2015; Hsiao et al., 2014; Jaspers‐Fayer et al., 2017; Macaulay et al., 1991; Murphy & Pichichero, 2002; Özen et al., 2019; Swedo et al., 2015; Talati et al., 2008), of which six focused on children (Bernstein et al., 2010; Frankovich et al., 2015; Jaspers‐Fayer et al., 2017; Murphy & Pichichero, 2002; Özen et al., 2019; Swedo et al., 2015). Only one of these 12 articles was suitable for meta‐analysis, although there did appear to be an association between OCD and LUTS based on the article content. A review of smaller studies of PANS/PANDAS suggests that urinary urgency, frequency, and enuresis are prominent clinical symptoms in children diagnosed with OCD that is categorized as PANS/PANDAS (Bernstein et al., 2010; Frankovich et al., 2015; Jaspers‐Fayer et al., 2017; Murphy & Pichichero, 2002; Swedo et al., 2015).

### 3.5 Analysis of heterogeneity

**FIGURE 5:**
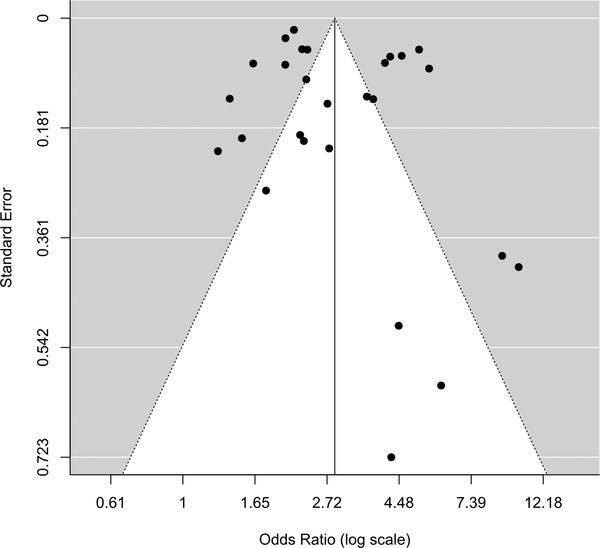
Funnel plot

We analyzed the heterogeneity of the studies for clinically significant anxiety among individuals diagnosed with LUTS. Visual inspection of the funnel plot (Figure 5) and the large value of I^2^ index and Cochran's Q test indicate high heterogeneity between studies. The Egger test was not statistically significant (*p* = .16), rejecting the null hypothesis that the funnel plot is asymmetrical. Nevertheless, the power of this method to detect bias is low with small numbers of studies. By visual inspection of the funnel plot, we observed an asymmetrical pattern mainly driven by five studies with large effect sizes but small sample sizes. Studies with small samples and negative effects were absent in our meta‐analysis. The funnel plot with the trim and fill method (Figure S3) suggests at least four studies with small samples, and negative effects were missing for a symmetrical funnel (Viechtbauer, 2007). However, after adjusting with the trim and fill method, I^2^ index did not decrease (I^2 ^= 97.86)

The majority of the eligible studies reported at least one domain of psychiatric disorders or urinary symptoms; therefore, most negative studies may be unpublished. There were between‐study variations in the methods of assessment for clinically significant anxiety and LUTS, which may have contributed to the heterogeneity of pooled estimates (see Sensitivity analysis).

### 3.6 Sensitivity analysis

We performed two sensitivity analyses. For the first analysis, we categorized clinically significant anxiety outcomes into three categories: (1) anxiety disorders based on the ICD code, (2) anxiety symptoms based on the HADS scale, and (3) anxiety symptoms based on other scales. We did not include PTSD in this analysis. For the HADS group, we estimated the OR = 3.41 (95% CI: 2.53,4.60, *p*< .001), Q = 356.01, I^2 ^= 97.4%; for the ICD‐9 group OR = 2.07 (95% CI: 1.72,2.49, *p* = .01), Q = 9.16, I^2 ^= 84.6%; and for the other scales OR = 3.03 (95% CI: 2.08,4.41, *p* < .001), Q = 67.79, I^2 ^= 88.9% (Figure S4). The ICD group had the lowest heterogeneity in comparison to the two other groups.

For the second analysis, we categorized LUTS outcomes into four categories: (1) IC, (2) OAB, (3) UI, and (4) other LUTS. For the IC group, we estimated OR = 2.41 (95% CI: 2.18,2.66, *p* = .37), Q = 1.98, I^2 ^= 0%; for the OAB group OR = 3.39 (95% CI: 2.53,4.55, *p* < 0.001), Q = 136.19, I^2^ = 94.1%; for the UI group OR = 2.82 (95% CI: 1.39,5.71, *p* = .004), Q = 29.6, I^2^ = 97.9%; and for the other LUTS group OR = 2.65 (95% CI: 1.57,4.47, *p* = .001), Q = 324.03, I^2^ = 98.8% (Figure S5). The IC group had the lowest heterogeneity; however, the estimate of I^2^ index was not significant due to the small sample size.

## 4 DISCUSSION

The results of this systematic review and meta‐analysis demonstrate reliable associations between clinically significant anxiety and LUTS. The OR for clinically significant anxiety amongst individuals with a LUTS diagnosis was 2.87 (95% CI: 2.38,3.46), or 3.01 (95% CI: 2.44,3.71) when excluding PTSD. The ORs were slightly lower in the few studies from the national registers (all from the Longitudinal Health Insurance Database in Taiwan), suggesting that the ORs from the case collection studies could be inflated due to ascertainment bias.

The OR for clinically significant anxiety among women with LUTS was slightly lower than men (3.14 vs. 3.52), although the number of included studies was small and the confidence intervals were overlapping. Further studies are required to confirm sex differences in LUTS among individuals with clinically significant anxiety and vice versa.

When examining the studies in which individuals were ascertained for clinically significant anxiety and then assessed for LUTS, findings were similar, with an OR of 2.87 (CI: 1.07,7.74). However, this result should be interpreted with caution since the sample size for this analysis was exceedingly small (*n* = 2), which is reflected in the wide confidence interval.

We found that the majority of studies examining the association between clinical anxiety and LUTS have failed to adjust for the use of selective serotonin reuptake inhibitors (SSRIs), which could increase the risk of bias. SSRIs are often prescribed to treat GAD, OCD, PD, social anxiety disorder, and PTSD (Bandelow, 2020; Bandelow et al., 2017). Multiple studies have shown that SSRIs can be associated with higher urinary frequency, urgency, and nocturia and induce urgent UI (Movig et al., 2002; Tsakiris et al., 2008).

In all analyses, we observed a large I^2^ index and Cochran's Q test, indicating high heterogeneity. Heterogeneity may be related to reporting bias, as supported by many studies plotting outside the funnel outline in the funnel plot (Figure 5). We analyzed the sensitivity of the results to the definition of the outcomes for clinically significant anxiety and LUTS. The results suggest that one of the leading causes of the high degree of heterogeneity in the results was the differing definitions of anxiety outcomes. The estimate of I^2^ index was the lowest when we categorized clinically significant anxiety based on ICD codes, which was the most well‐defined category. Although we estimated different values of I^2^ index for different dissections of outcomes in sensitivity analyses, the OR was larger than 2 for all the analyses, and none of the confidence intervals contained the value 1. Another interesting observation in the sensitivity analysis was that OAB had the highest OR among other LUTS categories.

In sum, there is compelling evidence for a strong association between clinically significant anxiety and LUTS, and it appears to be both bidirectional and clinically significant. Multiple studies were cross‐sectional, and the causality of the association in either direction could not be accurately determined using meta‐analysis. However, there were supports in the literature for a bidirectional association. A large longitudinal cohort study provides evidence for bidirectional relationships between LUTS and anxiety disorders (Huang et al., 2017), and a second longitudinal study showed that anxiety (measured using HADS) was both a risk factor for and a consequence of UI (Perry et al., 2006).

It was interesting to note that the association of anxiety disorders with LUTS appears to cross diagnostic categories. Previously, Weissman et al. posited a syndrome manifested by IC and PD (Weissman et al., 2004). However, our study shows that multiple anxiety disorder symptoms and multiple lower urinary tract symptoms show a similar relationship (Figure 3). Among psychiatric diagnoses, this relationship also extended to PTSD, which is characterized by prominent anxiety‐related symptoms although not classified in the DSM 5 anxiety disorder section due to its alternate phenomenology. We examined the result with and without PTSD because of the prominent anxiety symptoms that manifest in this disorder.

Our analyses also highlight that there are very significant gaps in the literature. First, studies of LUTS in individuals ascertained for an anxiety diagnosis, or clinically significant anxiety symptoms are rare. Studies in mental health settings are needed to elaborate on these associations so they can be more fully understood. Similarly, studies using national registers, where ascertainment and access issues are less confounding, could help us understand these associations, particularly since data are collected prospectively across a range of health care visits, related to both mental health and medical conditions, and additional demographic information can enrich our understanding of these relationships. Second, diagnostic criteria and definitions are not always consistent or clear in the existing studies. For example, there were few studies that made use of defined diagnoses and diagnostic criteria. Some studies used severity measures and others used standardized instruments for measuring general mental health. Therefore, we pooled the outcomes based on elevated levels of anxiety symptoms rather than formal anxiety diagnoses alone and defined the outcome as clinically significant anxiety. It is apparent that in order to understand the relationships with specific diagnoses, more studies are needed. Finally, and most strikingly, is the absence of studies of pediatric anxiety disorders. This is important to address for several reasons: (i) childhood LUTS is impairing and is predictive of adult LUTS; hence, treating LUTS (including associated anxiety) may have both immediate and long‐term impact; (ii) rates of LUTS increase with age; therefore, the study of pediatric age individuals may reveal differing findings and risks; (iii) furthermore, childhood‐onset anxiety disorders may differ from adult‐onset anxiety disorders, so, again, it may be inappropriate to generalize from adult studies to studies of youth. Given the strong findings in adults, we would argue that studies in children, as well as longitudinal studies, are critically needed.

In summary, our systematic review and meta‐analysis of the published literature indicate a significant association across multiple anxiety diagnoses, anxiety symptoms, and multiple lower urinary tract symptoms, regardless of the definition of clinically significant anxiety or LUTS. Careful assessment and evaluation of these often comorbid conditions is an important aspect of comprehensive clinical care, including referral to appropriate specialists, as indicated. These strong associations should also motivate the investigation of our significant knowledge gaps in this area, particularly the dearth of data in children. Undoubtedly, optimal treatment for LUTS needs to incorporate the psychiatric dimension, requiring a more precise understanding of these relationships. To the extent that urinary symptoms contribute to psychiatric symptoms, identification and treatment of active psychiatric symptoms should improve treatment response related to LUTS. To the extent that psychiatric symptoms contribute to urinary problems, treatment of urinary symptoms alone may provide an incomplete resolution of symptoms. It is also interesting to consider whether shared genetic or other factors may contribute to both urinary and psychiatric disorders and as such may ultimately provide insight into etiologic mechanisms. Understanding the causes of the observed relationships, be they biological or behavioral, is an important topic for future study.

## AUTHOR CONTRIBUTIONS

Behrang Mahjani had full access to all the data in the study and takes responsibility for the integrity of the data and the accuracy of the data analysis. Anita Batuure and Christina Gustavsson Mahjani contributed equally. *Study concept and design*: Olof Akre, Anita Batuure, Joseph D. Buxbaum, Dorothy E. Grice, Christina Gustavsson Mahjani, Magdalena Janecka, Lotta Renström Koskela, Behrang Mahjani. *Acquisition, analysis, or interpretation of data*: Olof Akre, Joseph D. Buxbaum, Dorothy E. Grice, Magdalena Janecka, Lotta Renström Koskela, Behrang Mahjani. *Drafting of the manuscript*: Anita Batuure, Joseph D. Buxbaum, Dorothy E. Grice, Christina Gustavsson Mahjani, Lotta Renström Koskela, Behrang Mahjani. *Critical revision of the manuscript for important intellectual content*: All authors. *Statistical analysis*: Behrang Mahjani. *Obtained funding*: Dorothy E. Grice. *Study supervision*: Olof Akre, Joseph D. Buxbaum, Dorothy E. Grice, Lotta Renström Koskela.

## Supporting information

The data that support the findings of this study are available from the corresponding author upon reasonable request.

## Data Availability

The data that support the findings of this study are available from the corresponding author upon reasonable request.
