# Supplementary material for: Systematic review and meta‐analysis identify significant relationships between clinical anxiety and lower urinary tract symptoms
Source: Brain Behav. 2021 Aug 17;11(9):e2268. doi: 10.1002/brb3.2268 (PMC8442597; doi:10.1002/brb3.2268)
Supplement: Supplementary file 1 — The data that support the findings of this study are available from the corresponding author upon reasonable request. [file BRB3-11-e2268-s001.docx]

**Systematic review and meta‐analysis identify significant relationships between clinically significant anxiety and lower urinary tract symptoms**

**Supplements:**

- Supplement 1: PRISMA checklist
- Supplement 2: Database Search Strategy
- Table S1. Characteristics of included studies (*n* = 94)
- Table S2. Characteristics of included studies in the meta-analysis (*n* = 23)
- Figure S1. Forest plot for clinically significant anxiety among individuals with LUTS, excluding the results for PTSD.
- Figure S2. Forest plot for LUTs among individuals with clinically significant anxiety.
- Figure S3. Funnel Plot, Trim and Fill method
- Figure S4. Sensitivity analysis of clinically significant anxiety outcomes
- Figure S5. Sensitivity analysis of LUTs outcomes

**Supplement 1: PRISMA 2009 Checklist**

| **Section/topic** | **#** | **Checklist item** | **Reported on page # or section #** |
| --- | --- | --- | --- |
| **TITLE** | | |  |
| Title | 1 | Identify the report as a systematic review, meta-analysis, or both. | First page |
| **ABSTRACT** | | |  |
| Structured summary | 2 | Provide a structured summary including, as applicable: background; objectives; data sources; study eligibility criteria, participants, and interventions; study appraisal and synthesis methods; results; limitations; conclusions and implications of key findings; systematic review registration number. | First page |
| **INTRODUCTION** | | |  |
| Rationale | 3 | Describe the rationale for the review in the context of what is already known. | Section 1 |
| Objectives | 4 | Provide an explicit statement of questions being addressed with reference to participants, interventions, comparisons, outcomes, and study design (PICOS). | Section 1 |
| **METHODS** | | |  |
| Protocol and registration | 5 | Indicate if a review protocol exists, if and where it can be accessed (e.g., Web address), and, if available, provide registration information including registration number. | Section 2.1 |
| Eligibility criteria | 6 | Specify study characteristics (e.g., PICOS, length of follow-up) and report characteristics (e.g., years considered, language, publication status) used as criteria for eligibility, giving rationale. | Section 2.1 |
| Information sources | 7 | Describe all information sources (e.g., databases with dates of coverage, contact with study authors to identify additional studies) in the search and date last searched. | Section 2.1 |
| Search | 8 | Present full electronic search strategy for at least one database, including any limits used, such that it could be repeated. | Section 2.1,  Supplement 2 |
| Study selection | 9 | State the process for selecting studies (i.e., screening, eligibility, included in systematic review, and, if applicable, included in the meta-analysis). | Section 2.1 |
| Data collection process | 10 | Describe method of data extraction from reports (e.g., piloted forms, independently, in duplicate) and any processes for obtaining and confirming data from investigators. | Section 2.4 |
| Data items | 11 | List and define all variables for which data were sought (e.g., PICOS, funding sources) and any assumptions and simplifications made. | Sections 2.2, 2.3 |
| Risk of bias in individual studies | 12 | Describe methods used for assessing risk of bias of individual studies (including specification of whether this was done at the study or outcome level), and how this information is to be used in any data synthesis. | Section 2.4 |
| Summary measures | 13 | State the principal summary measures (e.g., risk ratio, difference in means). | Section 2.4 |
| Synthesis of results | 14 | Describe the methods of handling data and combining results of studies, if done, including measures of consistency (e.g., I^2^) for each meta-analysis. | Section 2.4 |

| **Section/topic** | **#** | **Checklist item** | **Reported on page # or section #** |
| --- | --- | --- | --- |
| Risk of bias across studies | 15 | Specify any assessment of risk of bias that may affect the cumulative evidence (e.g., publication bias, selective reporting within studies). | Section 2.4 |
| Additional analyses | 16 | Describe methods of additional analyses (e.g., sensitivity or subgroup analyses, meta-regression), if done, indicating which were pre-specified. | Section 2.4 |
| **RESULTS** | | |  |
| Study selection | 17 | Give numbers of studies screened, assessed for eligibility, and included in the review, with reasons for exclusions at each stage, ideally with a flow diagram. | Section 3, Figure 2 |
| Study characteristics | 18 | For each study, present characteristics for which data were extracted (e.g., study size, PICOS, follow-up period) and provide the citations. | Table S1 and S2 |
| Risk of bias within studies | 19 | Present data on risk of bias of each study and, if available, any outcome level assessment (see item 12). | Figure 3-4 |
| Results of individual studies | 20 | For all outcomes considered (benefits or harms), present, for each study: (a) simple summary data for each intervention group (b) effect estimates and confidence intervals, ideally with a forest plot. | Section 3.1-3.4, Figure 3-4, Figure S1, S2 |
| Synthesis of results | 21 | Present results of each meta-analysis done, including confidence intervals and measures of consistency. | Figure 3,4, S1, S2 |
| Risk of bias across studies | 22 | Present results of any assessment of risk of bias across studies (see Item 15). | Section 3.5 and 3.6 |
| Additional analysis | 23 | Give results of additional analyses, if done (e.g., sensitivity or subgroup analyses, meta-regression [see Item 16]). | Figure 4, Figure S1-S5 |
| **DISCUSSION** | | |  |
| Summary of evidence | 24 | Summarize the main findings including the strength of evidence for each main outcome; consider their relevance to key groups (e.g., healthcare providers, users, and policy makers). | Section 4 |
| Limitations | 25 | Discuss limitations at study and outcome level (e.g., risk of bias), and at review-level (e.g., incomplete retrieval of identified research, reporting bias). | Section 4 |
| Conclusions | 26 | Provide a general interpretation of the results in the context of other evidence, and implications for future research. | Section 4 |
| **FUNDING** | | |  |
| Funding | 27 | Describe sources of funding for the systematic review and other support (e.g., supply of data); role of funders for the systematic review. | ACKNOWLEDGMENTS |

From: Moher D, Liberati A, Tetzlaff J, Altman DG, The PRISMA Group (2009). Preferred Reporting Items for Systematic Reviews and Meta-Analyses: The PRISMA Statement. PLoS Med 6(7): e1000097. doi:10.1371/journal.pmed1000097;

**Supplement 2. Database Search Strategy**

**PubMed**

((urinary) OR (lower urinary tract) OR (overactive bladder) OR (bladder Pain Syndrome) OR (interstitial cystitis) OR (incontinence)) AND ((phobias) OR (agoraphobia) OR (adjustment disorders) OR (phobic) OR (anxiety) OR (obsessive-compulsive) OR (panic disorder) OR (post-traumatic stress) OR (adjustment disorders) OR (selective mutism) OR (pediatric autoimmune neuropsychiatric disorders associated with streptococcal) OR (pediatric acute-onset neuropsychiatric syndrome)) AND (urinary[Title] OR lower urinary tract[Title] OR overactive bladder[Title] OR bladder pain syndrome[Title] OR interstitial cystitis[Title] OR incontinence[Title] OR phobic[Title] OR anxiety[Title] OR obsessive-compulsive[Title] OR panic disorder[Title] OR post-traumatic stress[Title] OR adjustment disorders[Title] OR agoraphobia[Title] OR phobias[Title] OR adjustment disorders[Title] OR selective mutism[Title] OR pediatric autoimmune neuropsychiatric disorders associated with streptococcal[Title] OR pediatric acute-onset neuropsychiatric syndrome[Title]) NOT rats[Title] NOT rat[Title] NOT dog[Title] NOT dogs[Title] NOT rabbits[Title] NOT rabbit[Title] NOT mice[Title]

Note: Similar strategies were used to search in Pysnet and CENTRAL (Cochrane Central Register of Controlled Trials (CENTRAL)).

**Google Scholar**

We used Google Scholar to find articles that PubMed, Psynet, and CENTRAL searches might have missed. We performed 12 searches using the keywords below. For each search, we screened the first 100 results to find any relevant articles that PubMed, PsycINFO, and Cochrane searches did not identify.

1. "obsessive-compulsive" "Interstitial cystitis"

2. "obsessive-compulsive" "overactive bladder"

3. "obsessive-compulsive" "urinary"

4. "Pediatric Autoimmune Neuropsychiatric Disorders Associated with Streptococcal" " Interstitial cystitis "

5. "Pediatric Autoimmune Neuropsychiatric Disorders Associated with Streptococcal" " overactive bladder "

6. "Pediatric Autoimmune Neuropsychiatric Disorders Associated with Streptococcal" "urinary"

7. "Pediatric Acute-onset Neuropsychiatric Syndrome" "Interstitial cystitis"

8. "Pediatric Acute-onset Neuropsychiatric Syndrome" "overactive bladder"

9. "Pediatric Acute-onset Neuropsychiatric Syndrome" "urinary"

10. "anxiety" "Interstitial cystitis"

11. "anxiety" "overactive bladder"

12. "anxiety" "urinary"

**Table S1.** Characteristics of included studies (*n* = 94)

| **Authors** | **Ref** | **LUTS type** | **Clinically significant anxiety type** | **Age** | **Country of Origin** | **Notes** | **Meta-analysis**  **1: yes/ 0:No** |
| --- | --- | --- | --- | --- | --- | --- | --- |
| (Abdul-Razzak et al., 2019) | (1) | OAB | Anxiety | Mean: 37.7 | Jordan |  | 1 |
| (Ahn et al., 2016) | (2) | OAB | OCD | Range: 20 - 86 | Korea | Females Only | 0 |
| (Asoglu, Selcuk, Cam, Cogendez, & Karateke, 2014) | (3) | Urinary Incontinence | Anxiety | Mean: 54.1 | Turkey |  | 0 |
| (Bernstein, Victor, Pipal, & Williams, 2010) | (4) | Urinary Urgency | PANDAS/OCD | Range: 6-14 | United States | Children | 0 |
| (Bogner, O'Donnell, de Vries, Northington, & Joo, 2011) | (5) | Urinary Incontinence | Social Phobia, Agoraphobia, Panic Disorder, OCD | Range: 30- 86 | United States |  | 0 |
| (Bogner, Gallo, Swartz, & Ford, 2005) | (6) | Urinary Incontinence | Anxiety | Range: 50+ | United States |  | 0 |
| (Bradley et al., 2014) | (7) | OAB | PTSD, Anxiety | Mean: 31.1 | United States | Females only | 1 |
| (Bradley et al., 2012) | (8) | OAB | PTSD, Anxiety | Range: 20- 67 | United States | Females only | 1 |
| (Bradley, Nygaard, Hillis, Torner, & Sadler, 2017) | (9) | Urinary Incontinence | PTSD | Range: Mean 38.7 | United States | Females only | 0 |
| (Breyer et al., 2014) | (10) | LUTS defined by ICD-9 codes | PTSD | Range: 18-64  Mean: 31.8 | United States |  | 1 |
| (Bruch, 2016) | (11) | Urinary tract infection | Anxiety | Mean: 45.76 | United States |  | 0 |
| (Cepeda, Reps, Sena, & Ochs-Ross, 2019) | (12) | IC/BPS | Anxiety (ICD-9 and ICD-10) | Mean IC: 50.87 Mean no IC: 47.47 | United States |  | 0 |
| (E. P. H. Choi, Lam, & Chin, 2016b) | (13) | LUTS | Anxiety | Range: 18+ | China |  | 0 |
| (W. S. Choi, Heo, Lee, & Son, 2017) | (14) | LUTS | Anxiety | Mean: 48.4 | Korea |  | 0 |
| (E. P. H. Choi, Lam, & Chin, 2014) | (15) | LUTS | Anxiety | Mean: 62.5 | China |  | 0 |
| (E. P. H. Choi, Lam, & Chin, 2016a) | (16) | LUTS | Anxiety | Mean: 62.5 | China |  | 0 |
| (Choo et al., 2007) | (17) | Urinary Incontinence | Anxiety | Range: 30-79 | Korea | Females only | 0 |
| (Chuang, Weng, Hsu, Huang, & Wu, 2015) | (18) | Bladder Pain Syndrome, Interstitial Cystitis | Anxiety | Mean: 46 | Taiwan | . | 1 |
| (Chung, Liu, Lin, & Chung, 2014) | (19) | Bladder Pain Syndrome, Interstitial Cystitis | Anxiety | Mean: 47.5 | Taiwan |  | 0 |
| (Clemens, Meenan, O’Keeffe Rosetti, Kimes, & Calhoun, 2008) | (20) | Interstitial Cystitis | Anxiety |  | United States | Females only | 1 |
| (Clemens, Brown, & Calhoun, 2008) | (21) | Interstitial Cystitis, Painful Bladder Syndrome | Panic Disorder |  | United States |  | 0 |
| (Clemens, Elliott, Suttorp, & Berry, 2012) | (22) | Interstitial Cystitis, Bladder Pain Syndrome | Panic Attacks |  | United States | Females only | 0 |
| (Coyne, Wein, et al., 2009) | (23) | Voiding, Storage,  Post micturition | Anxiety | Range: 40+ | United States |  | 1 |
| (Coyne, Kaplan, et al., 2009) | (24) | Voiding, Storage,  Post micturition | Anxiety | Range: 40+ | United States |  | 0 |
| (Coyne et al., 2011) | (25) | OAB | Anxiety |  | Sweden, UK |  | 0 |
| (Coyne et al., 2012) | (26) | Urinary Incontinence | Anxiety | Range: 40+ | Sweden, UK, US |  | 0 |
| (Drummond et al., 2012) | (27) | Urinary incontinence | OCD | Range: 19 - 70 | United Kingdom |  | 0 |
| (Dybowski, Löwe, & Brünahl, 2018) | (28) | Urinary Symptoms, PB/PPS | Anxiety | Mean: 49.3 | Germany |  | 0 |
| (Fan, Lin, Wu, Hong, & Chen, 2008) | (29) | Interstitial Cystitis | Anxiety | Range: 23-79  Mean: 50.2 | Taiwan |  | 0 |
| (Felde, Bjelland, & Hunskaar, 2012) | (30) | Urinary Incontinence | Anxiety | Range: 40-44  Mean: 42 | Norway | Female only | 1 |
| (Felde, Ebbesen, & Hunskaar, 2017) | (31) | Urinary Incontinence | Anxiety | Range: >20 | Norway |  | 0 |
| (Filce & Lavergne, 2013) | (32) | Urinary and Fecal Incontinence | Anxiety | Range: 11-18 | United States | Children | 0 |
| (Frankovich et al., 2015) | (33) | Urinary Frequency | Anxiety | Range: 3-17 | United States | Children | 0 |
| (Glover, Gannon, McLoughlin, & Emberton, 2004) | (34) | Mixed LUTS | Anxiety | Range: 39-89 | United Kingdom | Males only | 0 |
| (Alexander Von Gontard, Moritz, Thome-Granz, & Equit, 2015) | (35) | Incontinence | Anxiety | Mean: 6.2 | Germany | Children | 0 |
| (A. Von Gontard et al., 1998) | (36) | Urge Incontinence, Voiding Postponement | Anxiety, ICD - 10 | Range: 5-10.9 | Germany | Children | 0 |
| (Helfand et al., 2018) | (37) | Urinary Incontinence | Anxiety | Mean: 60.9 | United States | Male only | 0 |
| (Hsiao, Liao, Chen, Chang, & Lin, 2014) | (38) | OAB | Anxiety, OCD, Phobic Anxiety | Mean case: 53.2 Mean control: 52.3 | Taiwan | Females Only | 0 |
| (Lung-Cheng Huang et al., 2015) | (39) | LUTS | Anxiety | Case mean: 51.83 Control mean: 51.83 | Taiwan |  | 1 |
| (Huang, Wu, Ho, & Wang, 2017) | (40) | LUTS | Anxiety | Mean: 52.02 | Taiwan |  | 1 |
| (Jaspers-Fayer et al., 2017) | (41) | Urinary urgency | OCD | Range: 6-19 | Sweden | Children | 0 |
| (Joinson, Heron, & von Gontard, 2006) | (42) | Daytime Wetting | General anxiety | Range: 7-9 | United Kingdom | Children | 1 |
| (Katz et al., 2013) | (43) | IC/BPS | Anxiety | Mean: 52.58 | Canada, Sweden, US |  | 0 |
| (Kelleher et al., 2018) | (44) | OAB | Anxiety | Range: 18+ | United States |  | 0 |
| (Knight, Luft, Nakagawa, & Katzman, 2012) | (45) | Dry OAB | Anxiety | Range: 18-55  Case mean: 37.3 Control mean: 35.3 | United States | Female only | 0 |
| (Koh et al., 2015) | (46) | Storage, Voiding, Post micturition difficulties | Anxiety | Range: 40 + | Korea | Males only | 0 |
| (Kwon & Lee, 2014) | (47) | Urinary Incontinence | Anxiety | Range: 20+ | Korea |  | 0 |
| (H. Lai, Gardner, Vetter, & Andriole, 2015) | (48) | OAB, Interstitial Cystitis/Bladder Pain Syndrome | Anxiety | OAB mean: 53.7 IC/BPS mean: 44.8 Control mean: 54.2 | United States |  | 0 |
| (H. H. Lai, Rawal, Shen, & Vetter, 2016) | (49) | OAB, Urinary Incontinence | Anxiety | Case mean: 53.8 Control mean: 54.2 | United States |  | 1 |
| (H. H. Lai, Shen, Rawal, & Vetter, 2016) | (50) | OAB/Incontinence | Anxiety | Mean: 53.8 | United States |  | 0 |
| (H. H. Lai, Vetter, Jain, & Andriole, 2016) | (51) | OAB | Anxiety | Mean: 53.9 | United States |  | 0 |
| (Lagro-Janssen, Debruyne, & Van Weel, 1992) | (52) | Urinary Incontinence | Anxiety | Range: 20-65 | The Netherlands | Females only | 0 |
| (Lee et al., 2017) | (53) | OAB | Anxiety | Range: 40+ | China, Taiwan, South Korea |  | 0 |
| (R. Lim et al., 2018) | (54) | Stress Incontinence | Anxiety | Mean: 52.2 | Malaysia | Female only | 1 |
| (J. R. Lim, Bak, & Lee, 2007) | (55) | Mixed Incontinence, Stress Incontinence | Anxiety | MI mean: 47.3 | South Korea |  | 0 |
| (Macaulay, Stern, & Stanton, 1991) | (56) | Genuine stress incontinence, Detrusor instability, Sensory urgency | Anxiety | Mean: 46.6  Range: 15 - 79 | United Kingdom | Female only | 0 |
| (Martin et al., 2015) | (57) | Storage, Voiding | Anxiety | Mean: 55 | Australia | Male only | 1 |
| (Melotti, Juliato, Tanaka, & Riccetto, 2018) | (58) | OAB | Anxiety | Mean: 50.2 | Brazil |  | 0 |
| (Melville et al., 2002) | (59) | Urinary Incontinence (stress, urge, mixed) | Anxiety |  | United States | . | 0 |
| (Milsom, Kaplan, Coyne, Sexton, & Kopp, 2012) | (60) | OAB | Anxiety | Range: > 40 | United States |  | 1 |
| (Murphy & Pichichero, 2002) | (61) | Urinary urgency, urinary frequency | OCD | Range: 5-11 | United States | Children | 0 |
| (Nickel et al., 2010) | (62) | Interstitial Cystitis/Painful Bladder Syndrome | Anxiety | Mean: 49.64 | Canada | Female only | 0 |
| (Niemczyk et al., 2018) | (63) | Daytime Urinary Incontinence | Separation Anxiety Disorder, Specific Phobia, Social Phobia | Range: 5-13 | Germany | Children | 0 |
| (Norton, Bhat, & Stanton, 1990 | (64) | Urinary Incontinence | Anxiety | Mean: 41.2  Range: 41.2 - 48.5 | United Kingdom | Female only | 0 |
| (Oliver, Campigotto, Coplen, Traxel, & Austin, 2013) | (65) | Enuresis | Anxiety | Range: 6-17 | United States | Children | 0 |
| (Özen et al., 2019) | (66) | LUTS | Anxiety | Range: 6-7 | Turkey | Children | 0 |
| (Perry, McGrother, & Turner, 2006) | (67) | Urge Incontinence | Anxiety | Range: 40+ | United Kingdom | Females only | 1 |
| (Rapariz-González, Castro-Díaz, & Mejía-Rendón, 2014) | (68) | Urinary Symptoms, PB/PPS | Anxiety | Mean: 60.2 | Spain |  | 0 |
| (Rebassa et al., 2013) | (69) | Urinary incontinence | Anxiety | Range: 29+ | Spain |  | 1 |
| (Safarinejad, 2009) | (70) | Wet and dry OAB | Anxiety | Range: 15 - 55 | Iran | Female only | 1 |
| (Sakakibara et al., 2007) | (71) | Urinary Dysfunction | Anxiety | Mean: 37 | Japan |  | 0 |
| (Schast, Zderic, Richter, Berry, & Carr, 2008) | (72) | Voiding Dysfunction | ADHD | Mean: 57.2 | United States | Children | 0 |
| (Siddiqui et al., 2018) | (73) | LUTS | Anxiety | Mean: 56.7 | United States | Female only | 0 |
| (Siracusano et al., 2003) | (74) | Urinary Incontinence | Anxiety/nervousness | Range: 18-49.9 | Italy | Females only | 0 |
| (Sobański et al., 2016) | (75) | Urinary Frequency, Urinary Incontinence | Anxiety | Women mean: 33 Men mean: 32 | Poland |  | 0 |
| (Stach-Lempinen et al., 2003) | (76) | Urinary Incontinence | Anxiety | Range: 28-70 | Finland | Females only | 0 |
| (Stickley, Santini, & Koyanagi, 2017) | (77) | Urinary Incontinence | Anxiety | Range: 50+ | Ireland |  | 0 |
| (Swedo et al., 2015) | (78) | Urinary Frequency | Anxiety | Range: 3-7 | United States | Children | 0 |
| (Talati et al., 2008) | (79) | Interstitial Cystitis | Panic Disorder, Social Anxiety Disorder | Range: 18-65 | United States |  | 1 |
| (Teloken et al., 2006) | (80) | OAB | Anxiety | Range: 15-55 | Brazil |  | 0 |
| (Thu, Vetter, & Lai, 2019) | (81) | OAB IC/BPS | Anxiety (HADS-A) | Mean IC: 44.8 Mean OAB: 53.8 | United States |  | 0 |
| (Tzeng et al., 2019) | (82) | OAB | PTSD, Anxiety | Range: 20-59 | Taiwan | . | 1 |
| (D. M. J. Vrijens et al., 2017) | (83) | Urinary Frequency | Anxiety | Mean: 56.7 | Netherlands |  | 0 |
| (D. Vrijens et al., 2019) | (84) | OAB | Anxiety | Range: 18+ | Netherlands |  | 0 |
| (Wang, Liao, Liu, Sumarsono, & Cong, 2018) | (85) | LUTS | Anxiety | Range: 40+ | China |  | 0 |
| (Watson, Currie, Curran, & Jarvis, 2000) | (86) | Urinary Incontinence | Anxiety | Mean: 56 | United Kingdom |  | 0 |
| (Weissman et al., 2004) | (87) | Interstitial Cystitis | Panic Disorder | Range: 18-70 | United States |  | 1 |
| (Kuizenga-Wessel et al., 2018) | (88) | Functional defecation | Anxiety, ADHD | Range: 6-16 | Netherlands | Children | 0 |
| (Wolfe-Christensen, Veenstra, Kovacevic, Elder, & Lakshmanan, 2012) | (89) | Dysfunctional Voiding | Anxiety | Range: 4-16 | United States | Children | 0 |
| (Wu et al., 2017) | (90) | OAB | Anxiety | Median case: 27.5 Median control: 26.0 | China | Female only | 1 |
| (Yang et al., 2014) | (91) | LUTS | Anxiety | Range: 40+  Mean 61.7 | Korea |  | 0 |
| (Yazdany, Bhatia, & Reina, 2014) | (92) | Urinary Incontinence | Anxiety | Case mean 48.6 Control mean: 44.8 | United States | Females only | 0 |
| (Yoo, Kim, Kim, Oh, & Kim, 2011) | (93) | OAB | Anxiety | Range: 30+ | Korea |  | 1 |
| (Zink, Freitag, & von Gontard, 2008) | (94) | Urinary incontinence | Anxiety | Range: 5-6 | Germany | Children | 0 |

**Table S2.** Characteristics of included studies in the meta-analysis (*n* = 23)

| **Authors** | **Ref** | **Sample Size** | **LUTS type** | **Clinically significant type (scale/diagnosis)** | **Age** | **Country of Origin** | **Notes** | **Study design** |
| --- | --- | --- | --- | --- | --- | --- | --- | --- |
| (Abdul-Razzak et al., 2019) | (1) | Cases: 55  Controls: 129 | OAB | Anxiety (HADS ≥ 8) | Mean: 37.7 | Jordan | Hospital controls | Case-control |
| (Bradley et al., 2014) | (7) | Cases: 375  Controls: 1326 | OAB | PTSD (PCL-C),  Anxiety (GAD-7≥ 10) | Mean: 31.1 | United States | Females only | Nationwide cohort study of urogenital symptoms in female veterans |
| (Bradley et al., 2012) | (8) | Cases Stress UI: 183  Cases Mixed UI: 191  Comparison group: 334 | OAB | PTSD (PCL-C),  Anxiety (GAD-7≥ 10) | Range: 20-67 | United States | Females only | Nationwide cohort study of urogenital symptoms in female veterans |
| (Breyer et al., 2014) | (10) | Cases: 507952  Controls: 11237 | LUTS defined by ICD-9 codes | PTSD | Range: 18-64  Mean: 31.8 | United States |  | cohort study (veterans) |
| (Chuang et al., 2015) | (18) | Cases: 16185  Controls: 32370 | Bladder Pain Syndrome, Interstitial Cystitis | Anxiety (ICD-9) | Mean 46 | Taiwan |  | Cohort study, nationwide population |
| (Clemens, Meenan, et al., 2008) | (20) | Cases: 239  Controls: 717 | Interstitial Cystitis | Anxiety (ICD-9) |  | United States | Females only | Case-control from EpicCare electronic medical record |
| (Coyne, Wein, et al., 2009) | (23) | Men, Cases: 3433  Comparison group: 4097  Female, Cases: 4120  Comparison group: 4006 | Voiding, Storage,  Post micturition | Anxiety (HADS ≥ 8) | Range: 40+ | United States |  | Cross‐sectional population‐representative survey |
| (Felde et al., 2012) | (30) | Cases: 3789  Comparison group: 1391 | Urinary Incontinence | Anxiety (HADS ≥ 8) | Range: 40-44,  Mean 42 | Norway | Female only | Cross-sectional population-based survey |
| (Lung-Cheng Huang et al., 2015) | (39) | Cases: 22980  Controls: 45960 | LUTS | Anxiety (ICD-9) | Mean (cases): 51.83,  Mean (controls): 51.83 | Taiwan |  | Cohort study, nationwide population |
| (Huang et al., 2017) | (40) | Non-anxiety: 91444  Anxiety: 45707 | LUTS | Anxiety (ICD-9) | Mean: 52.02 | Taiwan |  | Cohort study, nationwide population |
| (Joinson et al., 2006) | (42) | Cases: 8213  Controls: 7570 | Daytime Wetting | General anxiety (DAWBA) | Range:7-9 | United Kingdom | Children | Population-based longitudinal study |
| (H. H. Lai, Rawal, et al., 2016) | (49) | Cases: 51  Controls: 30 | OAB, Urinary Incontinence | Anxiety (HADS ≥ 8) | Mean (cases): 53.8  Mean (controls): 54.2 | United States |  | Case-control |
| (R. Lim et al., 2018) | (54) | Cases:120  Comparison group: 145 | Stress Incontinence | Anxiety (EQ-5D) | Mean: 52.2 | Malaysia | Female only | Cross-sectional |
| (Martin et al., 2015) | (57) | Cases (voiding): 88  Controls: 642 | Storage, Voiding | Anxiety (GAD-7 ≥ 10) | Mean: 55 | Australia | Male only | Population-based prospective cohort study of randomly-selected men |
| (Milsom et al., 2012) | (60) | Men, cases 1512 comparison group: 1003  Female, cases: 3036  comparison group: 1456 | OAB | Anxiety (HADS ≥ 8) | Range: 40+ | United States |  | Population-based, cross-sectional Internet survey (EpiLUTS) |
| (Perry et al., 2006) | (67) | Cases: 1851  Controls: 10272 | Urge Incontinence | Anxiety (HADS ≥ 8) | Range: 40+ | United Kingdom | Females only | Cross-sectional (baseline)/longitudinal study |
| (Rebassa et al., 2013) | (69) | Cases: 162  Comparison group: 502 | Urinary incontinence | Anxiety (EG-5D) | Range: 29+ | Spain |  | Cross-sectional |
| (Safarinejad, 2009) | (70) | Cases: 1420  Comparison group: 6385 | Wet and dry OAB | Anxiety (not specified) | Range: 15 - 55 | Iran | Female only | Cross-sectional |
| (Talati et al., 2008) | (79) | PD: 219  SAD: 199  Controls: 102 | Interstitial Cystitis | Panic Disorder, Social Anxiety Disorder | Range: 18-65 | United States |  | Case-controls |
| (Tzeng et al., 2019) | (82) | Cases:811  Controls: 3244 | OAB | PTSD, Anxiety (ICD 9) | Range: 20-59 | Taiwan |  | Retrospective, matched-cohort design |
| (Weissman et al., 2004) | (87) | Cases: 67  Controls: 79 | Interstitial Cystitis | Panic Disorder | Range: 18-70 | United States |  | Case-control |
| (Wu et al., 2017) | (90) | Cases: 30  Comparison group: 25 | OAB | Anxiety (SAS ≥ 50) | Median (cases): 27.5  Median (control): 26.0 | China | Female only | Cross-sectional |
| (Yoo et al., 2011) | (93) | Cases: 458  Comparison group: 1542 | OAB | Anxiety (HADS ≥ 8) | Range>30+ | Korea |  | Cross-sectional telephone survey |

**Figure S1.** Forest plot for clinically significant anxiety among individuals with LUTS, excluding the results for PTSD


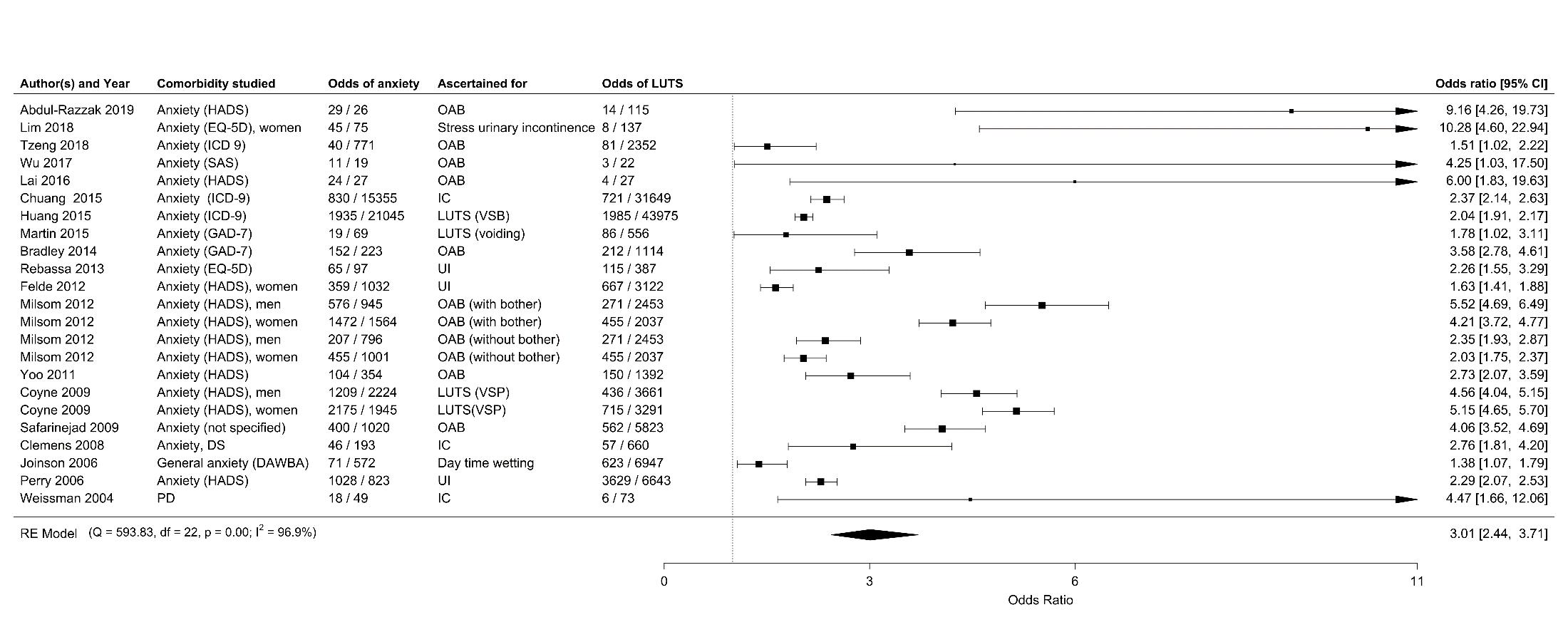


PD: panic disorder, OAB: overactive bladder, IC: interstitial cystitis, UI: urinary incontinence, LUTS: lower urinary tract symptoms, VSP: voiding, storage, postmicturition, VSB: voiding, storage, benign prostatic hyperplasia, EQ-5D: EuroQol: standardized instrument for measuring generic health status, HADS: Hospital Anxiety and Depression Scale, SAS: The Zung Self-Rating Anxiety Scale, GAD-7: General Anxiety Disorder 7-item, DAWBA: Development And Well-Being Assessment, ICD 9: International Classification of Diseases code version 9, DS: dissociative and somatoform disorders. P is the p-value for I^2^.

**Figure S2.** Forest plot for LUTs among individuals with clinically significant anxiety.


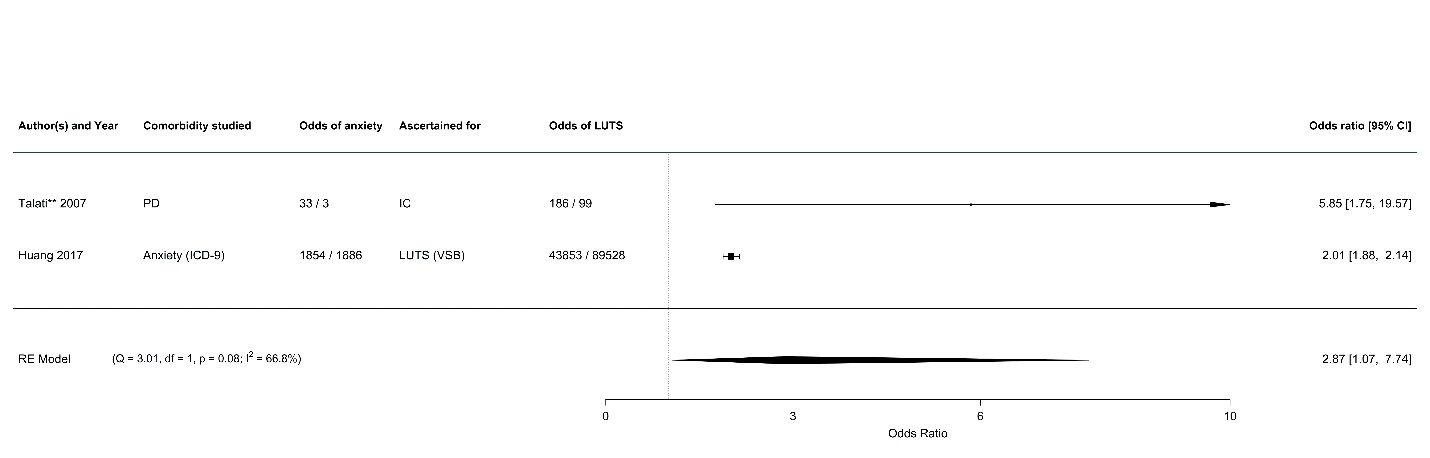


PD: panic disorder, IC: interstitial cystitis, LUTS: lower urinary tract symptoms, VSB: voiding, storage, benign prostatic hyperplasia, ICD 9: International Classification of Diseases code version 9. **: The number of missing values in this study were not reported. Including only 1 analysis from Talati 2007. P is the p-value for I^2^.

**Figure S3.** Funnel Plot, Trim and Fill method

**
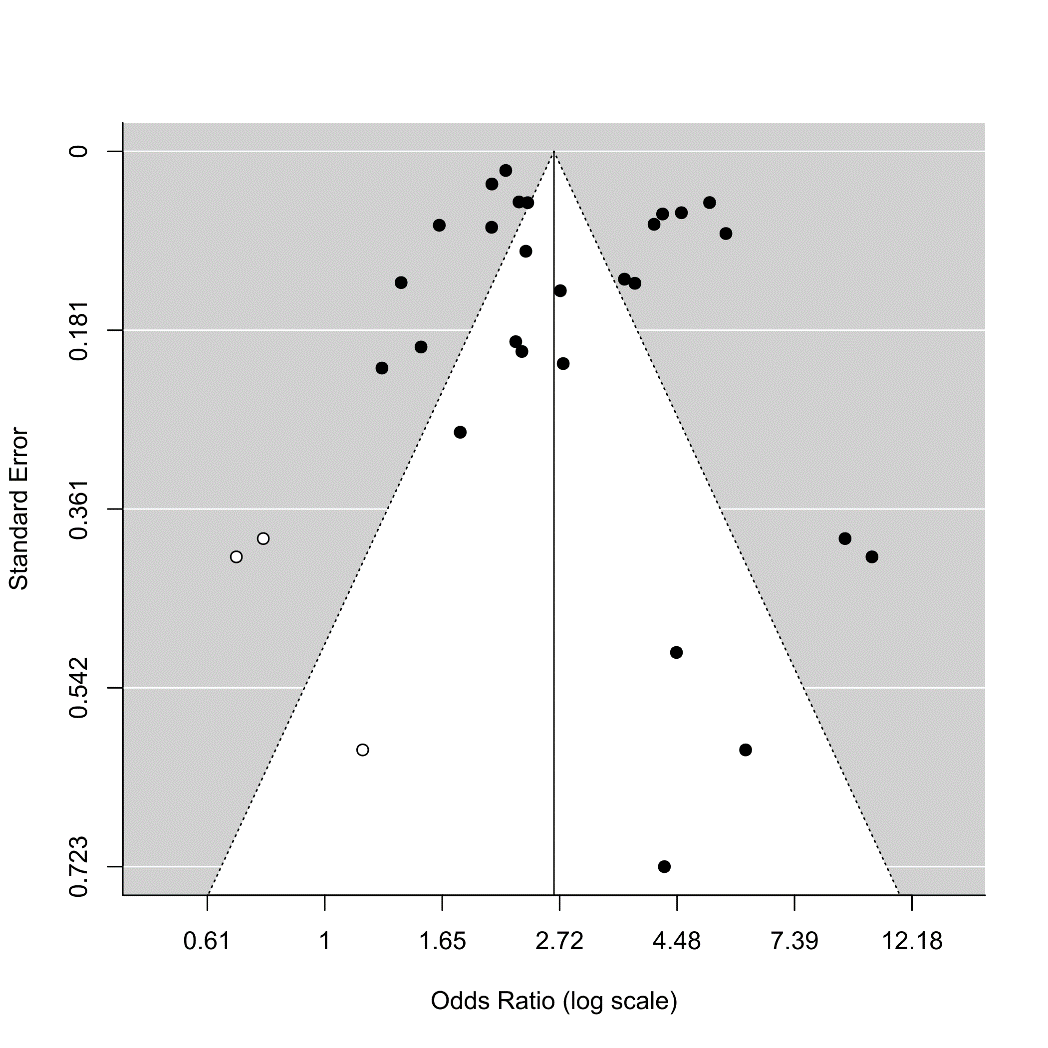
**

**Figure S4.** Sensitivity analysis of clinically significant anxiety outcomes


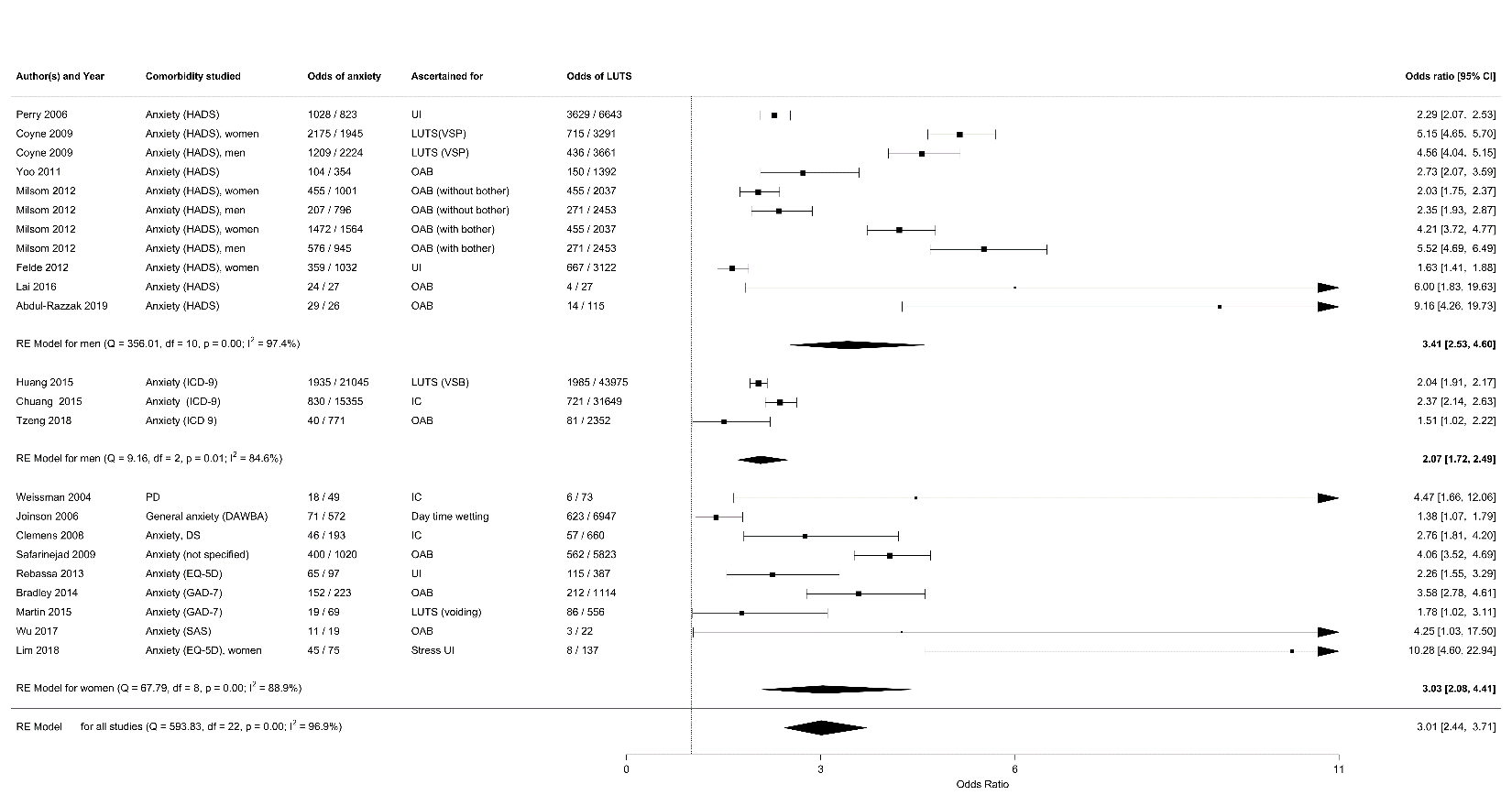


PD: panic disorder, OAB: overactive bladder, IC: interstitial cystitis, UI: urinary incontinence, LUTS: lower urinary tract symptoms, VSP: voiding, storage, postmicturition, , VSB: voiding, storage, benign prostatic hyperplasia, EQ-5D: EuroQol: standardized instrument for measuring generic health status, HADS: Hospital Anxiety and Depression Scale, SAS: The Zung Self-Rating Anxiety Scale, GAD-7: General Anxiety Disorder 7-item, DAWBA: Development And Well-Being Assessment, ICD 9: International Classification of Diseases code version 9, DS: dissociative and somatoform disorders. P is the p-value for I^2^.

**Figure S5.** Sensitivity analysis of LUTs outcomes

**
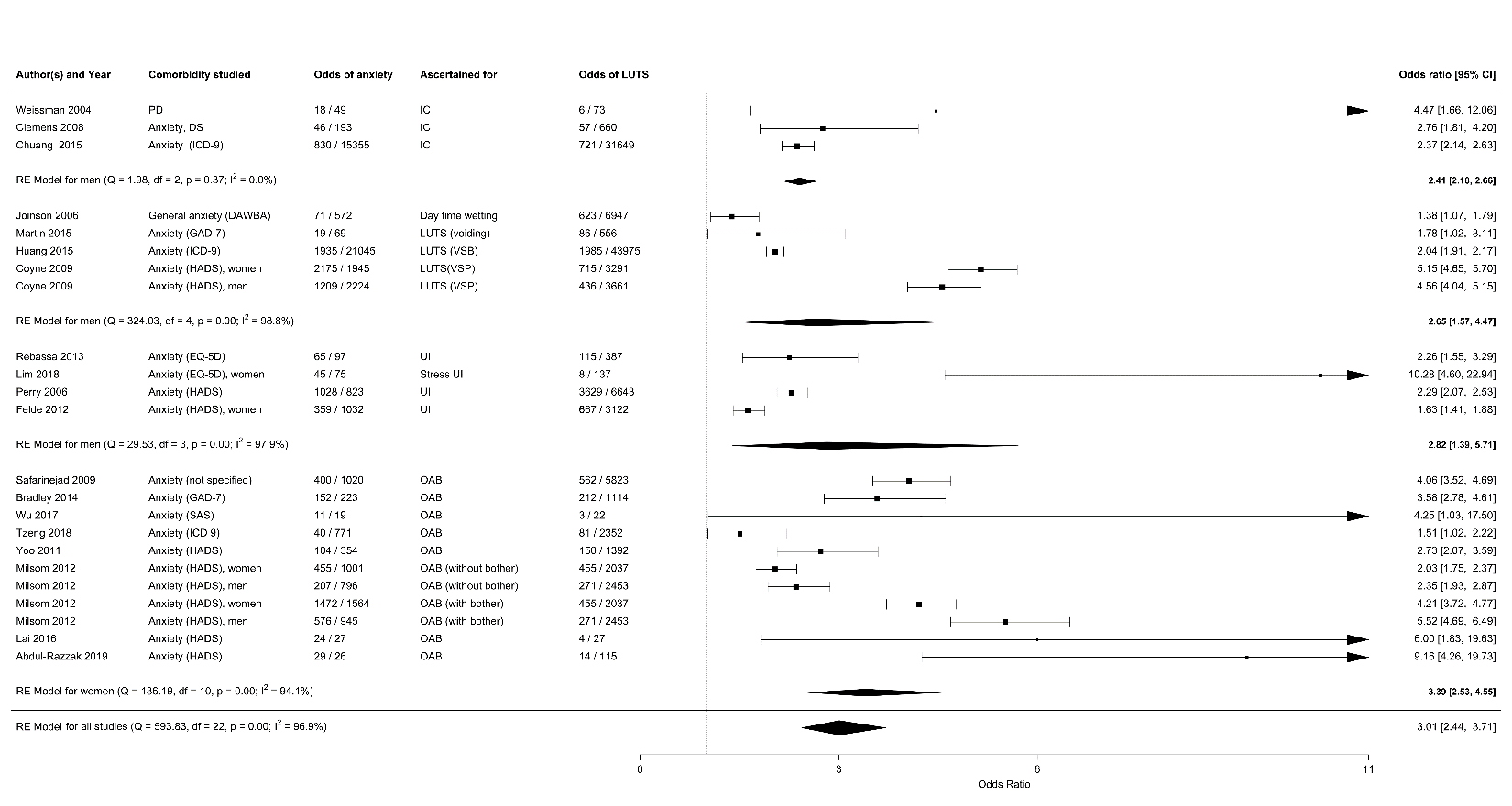
**

PD: panic disorder, OAB: overactive bladder, IC: interstitial cystitis, UI: urinary incontinence, LUTS: lower urinary tract symptoms, VSP: voiding, storage, postmicturition, VSB: voiding, storage, benign prostatic hyperplasia, EQ-5D: EuroQol: standardized instrument for measuring generic health status, HADS: Hospital Anxiety and Depression Scale, SAS: The Zung Self-Rating Anxiety Scale, GAD-7: General Anxiety Disorder 7-item, DAWBA: Development And Well-Being Assessment, ICD 9: International Classification of Diseases code version 9, DS: dissociative and somatoform disorders. P is the p-value for I^2^.

**References:**

1. Abdul-Razzak KK, Alshogran OY, Altawalbeh SM, et al.: Overactive bladder and associated psychological symptoms: A possible link to vitamin D and calcium. Neurourol Urodyn 2019;

2. Ahn KS, Hong HP, Kweon HJ, et al.: Correlation between overactive bladder syndrome and obsessive compulsive disorder in women. Korean J Fam Med 2016; 37:25–30

3. Asoglu MR, Selcuk S, Cam C, et al.: Effects of urinary incontinence subtypes on women's quality of life (including sexual life) and psychosocial state. Eur J Obstet Gynecol Reprod Biol 2014; 176:187–190

4. Bernstein GA, Victor AM, Pipal AJ, et al.: Comparison of Clinical Characteristics of Pediatric Autoimmune Neuropsychiatric Disorders Associated with Streptococcal Infections and Childhood Obsessive-Compulsive Disorder. J Child Adolesc Psychopharmacol 2010; 20:333–340

5. Bogner HR, O'Donnell AJ, de Vries HF, et al.: The temporal relationship between anxiety disorders and urinary incontinence among community-dwelling adults. J Anxiety Disord 2011; 25:203–208

6. Bogner HR, Gallo JJ, Swartz KL, et al.: Anxiety Disorders and Disability Secondary to Urinary Incontinence among Adults over Age 50. Int J Psychiatry Med 2005; 32:141–154

7. Bradley CS, Hillis SL, Johnson S, et al.: Overactive bladder and mental health symptoms in recently deployed female veterans. J Urol 2014; 191:1327–1332

8. Bradley CS, Nygaard IE, Mengeling MA, et al.: Urinary incontinence, depression and posttraumatic stress disorder in women veterans. Am J Obstet Gynecol 2012; 206:502.e1-502.e8

9. Bradley CS, Nygaard IE, Hillis SL, et al.: Longitudinal associations between mental health conditions and overactive bladder in women veterans. Am J Obstet Gynecol 2017; 217:430.e1-430.e8

10. Breyer BN, Cohen BE, Bertenthal D, et al.: Lower urinary tract dysfunction in male Iraq and Afghanistan war veterans: Association with mental health disorders: A population-based cohort study. Urology 2014; 83:312–319

11. Bruch JD: Intestinal infection associated with future onset of an anxiety disorder: Results of a nationally representative study. Brain Behav Immun 2016; 57:222–226

12. Cepeda MS, Reps J, Sena AG, et al.: Risk factors for interstitial cystitis in the general population and in individuals with depression. Int Neurourol J 2019; 23:40–45

13. Choi EPH, Lam CLK, Chin WY: Mental health of Chinese primary care patients with lower urinary tract symptoms. Psychol Heal Med 2016; 21:113–127

14. Choi WS, Heo NJ, Lee YJ, et al.: Factors that influence lower urinary tract symptom (LUTS)-related quality of life (QoL) in a healthy population. World J Urol 2017; 35:1783–1789

15. Choi EPH, Lam CLK, Chin WY: The health-related quality of life of Chinese patients with lower urinary tract symptoms in primary care. Qual Life Res 2014; 23:2723–2733

16. Choi EPH, Lam CLK, Chin WY: Mental Health Mediating the Relationship Between Symptom Severity and Health-Related Quality of Life in Patients with Lower Urinary Tract Symptoms. LUTS Low Urin Tract Symptoms 2016; 8:141–149

17. Choo MS, Ku JH, Oh SJ, et al.: Prevalence of urinary incontinence in Korean women: An epidemiologic survey. Int Urogynecol J 2007; 18:1309–1315

18. Chuang YC, Weng SF, Hsu YW, et al.: Increased risks of healthcare-seeking behaviors of anxiety, depression and insomnia among patients with bladder pain syndrome/interstitial cystitis: a nationwide population-based study. Int Urol Nephrol 2015; 47:275–281

19. Chung K-H, Liu S-P, Lin H-C, et al.: Bladder pain syndrome/interstitial cystitis is associated with anxiety disorder. Neurourol Urodyn 2014; 33:101–105

20. Clemens JQ, Meenan RT, O'Keeffe Rosetti MC, et al.: Case-Control Study of Medical Comorbidities in Women With Interstitial Cystitis. J Urol 2008; 179:2222–2225

21. Clemens JQ, Brown SO, Calhoun EA: Mental Health Diagnoses in Patients With Interstitial Cystitis/Painful Bladder Syndrome and Chronic Prostatitis/Chronic Pelvic Pain Syndrome: A Case/Control Study. J Urol 2008; 180:1378–1382

22. Clemens JQ, Elliott MN, Suttorp M, et al.: Temporal ordering of interstitial cystitis/bladder pain syndrome and non-bladder conditions. Urology 2012; 80:1227–1232

23. Coyne KS, Wein AJ, Tubaro A, et al.: The burden of lower urinary tract symptoms: Evaluating the effect of LUTS on health-related quality of life, anxiety and depression. BJU Int 2009; 103:4–11

24. Coyne KS, Kaplan SA, Chapple CR, et al.: Risk factors and comorbid conditions associated with lower urinary tract symptoms: EpiLUTS. BJU Int 2009; 103:24–32

25. Coyne KS, Sexton CC, Kopp ZS, et al.: The impact of overactive bladder on mental health, work productivity and health-related quality of life in the UK and Sweden: Results from EpiLUTS. BJU Int 2011; 108:1459–1471

26. Coyne KS, Kvasz M, Ireland AM, et al.: Urinary incontinence and its relationship to mental health and health-related quality of life in men and women in Sweden, the United Kingdom, and the United States. Eur Urol 2012; 61:88–95

27. Drummond LM, Boschen MJ, Cullimore J, et al.: Physical complications of severe, chronic obsessive-compulsive disorder: A comparison with general psychiatric inpatients. Gen Hosp Psychiatry 2012; 34:618–625

28. Dybowski C, Löwe B, Brünahl C: Predictors of pain, urinary symptoms and quality of life in patients with chronic pelvic pain syndrome (CPPS): A prospective 12-month follow-up study. J Psychosom Res 2018; 112:99–106[cited 2019 Aug 8]

29. Fan Y-H, Lin ATL, Wu H-M, et al.: Psychological profile of Taiwanese interstitial cystitis patients. . Int J Urol 2008; 15:416–8[cited 2019 Aug 8]

30. Felde G, Bjelland I, Hunskaar S: Anxiety and depression associated with incontinence in middle-aged women: A large Norwegian cross-sectional study. Int Urogynecol J 2012; 23:299–306

31. Felde G, Ebbesen MH, Hunskaar S: Anxiety and depression associated with urinary incontinence. A 10-year follow-up study from the Norwegian HUNT study (EPINCONT). Neurourol Urodyn 2017; 36:322–328

32. Filce HG, Lavergne LC: The impact of a 1-week residential program on anxiety in adolescents with incontinence: A quasi-experimental study. J Wound, Ostomy Cont Nurs 2013; 40:185–192

33. Frankovich J, Thienemann M, Pearlstein J, et al.: Multidisciplinary Clinic Dedicated to Treating Youth with Pediatric Acute-Onset Neuropsychiatric Syndrome: Presenting Characteristics of the First 47 Consecutive Patients. J Child Adolesc Psychopharmacol 2015; 25:38–47

34. Glover L, Gannon K, McLoughlin J, et al.: Men's experiences of having lower urinary tract symptoms: Factors relating to bother. BJU Int 2004; 94:563–567

35. Von Gontard A, Moritz AM, Thome-Granz S, et al.: Abdominal pain symptoms are associated with anxiety and depression in young children. Acta Paediatr Int J Paediatr 2015; 104:1156–1163

36. Von Gontard A, Lettgen B, Olbing H, et al.: Behavioural problems in children with urge incontinence and voiding postponement: a comparison of a paediatric and child psychiatric sample. BJU Int 1998; 81:100–106

37. Helfand BT, Smith AR, Lai HH, et al.: Prevalence and Characteristics of Urinary Incontinence in a Treatment Seeking Male Prospective Cohort: Results from the LURN Study. J Urol 2018; 200:397–404

38. Hsiao SM, Liao SC, Chen CH, et al.: Psychometric assessment of female overactive bladder syndrome and antimuscarinics-related effects. Maturitas 2014; 79:428–434

39. Lung-Cheng Huang C, Ho CH, Weng SF, et al.: The association of healthcare seeking behavior for anxiety and depression among patients with lower urinary tract symptoms: A nationwide population-based study. Psychiatry Res 2015; 226:247–251

40. Huang CLC, Wu MP, Ho CH, et al.: The bidirectional relationship between anxiety, depression, and lower urinary track symptoms: A nationwide population-based cohort study. J Psychosom Res 2017; 100:77–82

41. Jaspers-Fayer F, Chan E, Ellwyn R, et al.: Prevalence of Acute-Onset Subtypes in Pediatric Obsessive-Compulsive Disorder. J Child Adolesc Psychopharmacol 2017; 27:332–341

42. Joinson C, Heron J, von Gontard A: Psychological Problems in Children With Daytime Wetting. Pediatrics 2006; 118:1985–1993

43. Katz L, Tripp DA, Nickel JC, et al.: Disability in women suffering from interstitial cystitis/bladder pain syndrome. BJU Int 2013; 111:114–121

44. Kelleher C, Chapple C, Johnson N, et al.: Development of an overactive bladder assessment tool (BAT): A potential improvement to the standard bladder diary. Neurourol Urodyn 2018; 37:1701–1710

45. Knight S, Luft J, Nakagawa S, et al.: Comparisons of pelvic floor muscle performance, anxiety, quality of life and life stress in women with dry overactive bladder compared with asymptomatic women. BJU Int 2012; 109:1685–1689

46. Koh JS, Ko HJ, Wang SM, et al.: The relationship between depression, anxiety, somatization, personality and symptoms of lower urinary tract symptoms suggestive of benign prostatic hyperplasia. Psychiatry Investig 2015; 12:268–273

47. Kwon CS, Lee JH: Prevalence, risk factors, quality of life, and health-care seeking behaviors of female urinary incontinence: Results from the 4th Korean national health and nutrition examination survey VI (2007-2009). Int Neurourol J 2014; 18:31–36

48. Lai H, Gardner V, Vetter J, et al.: Correlation between psychological stress levels and the severity of overactive bladder symptoms. BMC Urol 2015; 15

49. Lai HH, Rawal A, Shen B, et al.: The Relationship Between Anxiety and Overactive Bladder or Urinary Incontinence Symptoms in the Clinical Population. Urology 2016; 98:50–57

50. Lai HH, Shen B, Rawal A, et al.: The relationship between depression and overactive bladder/urinary incontinence symptoms in the clinical OAB population. BMC Urol 2016; 16:1–8

51. Lai HH, Vetter J, Jain S, et al.: Systemic Nonurological Symptoms in Patients with Overactive Bladder. J Urol 2016; 196:467–472

52. Lagro-Janssen ALM, Debruyne FMJ, Van Weel C: Psychological Aspects of Female Urinary Incontinence in General Practice. Br J Urol 1992; 70:499–502

53. Lee KS, Yoo TK, Liao L, et al.: Association of lower urinary tract symptoms and OAB severity with quality of life and mental health in China, Taiwan and South Korea: Results from a cross-sectional, population-based study. BMC Urol 2017; 17

54. Lim R, Liong ML, Leong WS, et al.: The Impact of Stress Urinary Incontinence on Individual Components of Quality of Life in Malaysian Women. Urology 2018; 112:38–45

55. Lim JR, Bak CW, Lee JB: Comparison of anxiety between patients with mixed incontinence and those with stress urinary incontinence. Scand J Urol Nephrol 2007; 41:403–406

56. Macaulay AJ, Stern RS, Stanton SL: Psychological aspects of 211 female patients attending a urodynamic unit. J Psychosom Res 1991; 35:1–10

57. Martin S, Vincent A, Taylor A., et al.: Lower urinary tract symptoms, depression, anxiety and systemic inflammatory factors in men: A population-based cohort study. PLoS One 2015; 10:no pagination

58. Melotti IGR, Juliato CRT, Tanaka M, et al.: Severe depression and anxiety in women with overactive bladder. Neurourol Urodyn 2018; 37:223–228

59. Melville JL, Walker E, Katon W, et al.: Prevalence of comorbid psychiatric illness and its impact on symptom perception, quality of life, and functional status in women with urinary incontinence. Am J Obstet Gynecol 2002; 187:80–87

60. Milsom I, Kaplan SA, Coyne KS, et al.: Effect of bothersome overactive bladder symptoms on health-related quality of life, anxiety, depression, and treatment seeking in the United States: Results from EpiLUTS. Urology 2012; 80:90–96

61. Murphy M., Pichichero M.: Prospective identification and treatment of children with pediatric autoimmune neuropsychiatric disorder associated with group A streptococcal infection (PANDAS). Arch Pediatr Adolesc Med 2002; 156:356–361

62. Nickel JC, Tripp DA, Pontari M, et al.: Psychosocial Phenotyping in Women With Interstitial Cystitis/Painful Bladder Syndrome: A Case Control Study. J Urol 2010; 183:167–172

63. Niemczyk J, Equit M, Rieck K, et al.: EEG measurement of emotion processing in children with daytime urinary incontinence. Z Kinder Jugendpsychiatr Psychother 2018; 46:336–341

64. Norton KRW, Bhat A V., Stanton SL: Psychiatric aspects of urinary incontinence in women attending an outpatient urodynamic clinic. Br Med J 1990; 301:271–272

65. Oliver JL, Campigotto MJ, Coplen DE, et al.: Psychosocial comorbidities and obesity are associated with lower urinary tract symptoms in children with voiding dysfunction. J Urol 2013; 190:1511–1515

66. Özen MA, Mutluer T, Necef I, et al.: The overlooked association between lower urinary tract dysfunction and psychiatric disorders: a short screening test for clinical practice. J Pediatr Urol 2019;

67. Perry S, McGrother CW, Turner K: An investigation of the relationship between anxiety and depression and urge incontinence in women: Development of a psychological model. Br J Health Psychol 2006; 11:463–482

68. Rapariz-González M, Castro-Díaz D, Mejía-Rendón D: Evaluation of the impact of the urinary symptoms on quality of life of patients with painful bladder syndrome/chronic pelvic pain and radiation cystitis: EURCIS study. Actas Urológicas Españolas (English Ed 2014; 38:224–231

69. Rebassa M, Taltavull JM, Gutiérrez C, et al.: Urinary incontinence in Mallorcan women: Prevalence and quality of life. Actas Urológicas Españolas (English Ed 2013; 37:354–361

70. Safarinejad MR: Prevalence of the overactive bladder among Iranian women based on the International Continence Society definition: A population-based study. Int Urol Nephrol 2009; 41:35–45

71. Sakakibara R, Uchiyama T, Awa Y, et al.: Psychogenic urinary dysfunction: A uro-neurological assessment. Neurourol Urodyn 2007; 26:518–524

72. Schast AP, Zderic SA, Richter M, et al.: Quantifying demographic, urological and behavioral characteristics of children with lower urinary tract symptoms. J Pediatr Urol 2008; 4:127–133

73. Siddiqui NY, Wiseman JB, Cella D, et al.: Mental Health, Sleep and Physical Function in Treatment Seeking Women with Urinary Incontinence. J Urol 2018; 200:848–855

74. Siracusano S, Pregazzi R, D’Aloia G, et al.: Prevalence of urinary incontinence in young and middle-aged women in an Italian urban area. Eur J Obstet Gynecol Reprod Biol 2003; 107:201–204

75. Sobański J, Skalski M, Gołąbek T, et al.: Occurrence of selected lower urinary tract symptoms in patients of a day hospital for neurotic disorders. Psychiatr Pol 2016; 50:1181–1205

76. Stach-Lempinen B, Hakala AL, Laippala P, et al.: Severe depression determines quality of life in urinary incontinent women. Neurourol Urodyn 2003; 22:563–568

77. Stickley A, Santini ZI, Koyanagi A: Urinary incontinence, mental health and loneliness among community-dwelling older adults in Ireland. BMC Urol 2017; 17

78. Swedo SE, Seidlitz J, Kovacevic M, et al.: Clinical Presentation of Pediatric Autoimmune Neuropsychiatric Disorders Associated with Streptococcal infections in Research and Community Settings. J Child Adolesc Psychopharmacol 2015; 25:26–30

79. Talati A, Ponniah K, Strug LJ, et al.: Panic Disorder, Social Anxiety Disorder, and a Possible Medical Syndrome Previously Linked to Chromosome 13. Biol Psychiatry 2008; 63:594–601

80. Teloken C, Caraver F, Weber F., et al.: Overactive Bladder: Prevalence and Implications in Brazil. Eur Urol 2006; 49:1087–1092

81. Thu JHL, Vetter J, Lai HH: The Severity and Distribution of Nonurologic Pain and Urogenital Pain in Overactive Bladder are Intermediate Between Interstitial Cystitis and Controls. Urology 2019;

82. Tzeng NS, Chang HA, Chung CH, et al.: Risk of psychiatric disorders in overactive bladder syndrome: A nationwide cohort study in Taiwan. J Investig Med 2019; 67:312–318

83. Vrijens DMJ, Drossaerts JMAFL, Rademakers K, et al.: Associations of Psychometric Affective Parameters with Urodynamic Investigation for Urinary Frequency. LUTS Low Urin Tract Symptoms 2017; 9:166–170

84. Vrijens D, Marcelissen T, Drossaerts J, et al.: Self-consciousness/awareness and bladder sensations: Comparative study of overactive bladder patients and healthy volunteers. LUTS Low Urin Tract Symptoms 2019; 11:3–7

85. Wang JY, Liao L, Liu M, et al.: Epidemiology of lower urinary tract symptoms in a cross-sectional, population-based study the status in China. Med (United States) 2018; 97

86. Watson AJS, Currie I, Curran S, et al.: A prospective study examining the association between the symptoms of anxiety and depression and severity of urinary incontinence. Eur J Obstet Gynecol Reprod Biol 2000; 88:7–9

87. Weissman MM, Gross R, Fyer A, et al.: Interstitial Cystitis and Panic Disorder: A Potential Genetic Syndrome. Arch Gen Psychiatry 2004; 61:273–279[cited 2019 Aug 7]

88. Kuizenga-Wessel S, Koppen IJN, Vriesman MH, et al.: Attention Deficit Hyperactivity Disorder and Functional Defecation Disorders in Children. J Pediatr Gastroenterol Nutr 2018; 66:244–249

89. Wolfe-Christensen C, Veenstra AL, Kovacevic L, et al.: Psychosocial difficulties in children referred to pediatric urology: A closer look. Urology 2012; 80:907–913

90. Wu P, Chen Y, Zhao J, et al.: Urinary microbiome and psychological factors in women with Overactive bladder. Front Cell Infect Microbiol 2017; 7

91. Yang YJ, Koh JS, Ko HJ, et al.: The influence of depression, anxiety and somatization on the clinical symptoms and treatment response in patients with symptoms of lower urinary tract symptoms suggestive of benign prostatic hyperplasia. J Korean Med Sci 2014; 29:1145–1151

92. Yazdany T, Bhatia N, Reina A: Association of depression and anxiety in underserved women with and without urinary incontinence. Female Pelvic Med Reconstr Surg 2014; 20:349–353

93. Yoo ES, Kim BS, Kim DY, et al.: The impact of overactive bladder on health-related quality of life, sexual life and psychological health in Korea. Int Neurourol J 2011; 15:143–151

94. Zink S, Freitag CM, von Gontard A: Behavioral Comorbidity Differs in Subtypes of Enuresis and Urinary Incontinence. J Urol 2008; 179:295–298
